# Intracranial stenting as a bail-out option for posthemorrhagic cerebral vasospasm: a single-center experience with long-term follow-up

**DOI:** 10.1186/s12883-022-02862-4

**Published:** 2022-09-15

**Authors:** Ali Khanafer, Alexandru Cimpoca, Pervinder Bhogal, Hansjörg Bäzner, Oliver Ganslandt, Hans Henkes

**Affiliations:** 1 Neuroradiologische Klinik, Klinikum Stuttgart, Kriegsbergstraße 60, 70174 Stuttgart, Germany; 2 Department of Interventional Neuroradiology, The Royal London Hospital, London, UK; 3 Neurologische Klinik, Klinikum Stuttgart, Stuttgart, Germany; 4 Neurochirurgische Klinik, Klinikum Stuttgart, Stuttgart, Germany; 5 Medizinische Fakultät, Universität Duisburg-Essen, Essen, Germany

**Keywords:** Subarachnoid hemorrhage, Posthemorrhagic cerebral vasospasm, Endovascular treatment, Self-expanding stent

## Abstract

**Background:**

Cerebral vasospasm (CVS) is a leading cause of morbidity and mortality in patients after aneurysmal subarachnoid hemorrhage (aSAH). Endovascular treatment, including intraarterial infusion of drugs with vasodilation effects, and balloon- and stentriever angioplasty, are helpful but may achieve only short-term effects. There is a clinical need for long-lasting treatment of refractory recurrent vasospasm. We report our experience in stent implantation as a treatment for recurrent severe post-SAH vasospasm.

**Methods:**

A retrospective analysis of our institutional database of 883 patients with SAH, managed between January 2010 and December 2021, was performed. Six patients were identified as having received intracranial stenting in the context of post-SAH cerebral vasospasm. All patients were initially treated with intra-arterial infusion of nimodipine and/or milrinone. Self-expanding intracranial stents were implanted during endovascular aneurysm treatment to enable access despite impaired perfusion (Group 1) or as a bail-out strategy after failed intraarterial drug infusion or mechanical treatment (Group 2). All stented patients received dual antiplatelet therapy (DAPT) for 6 months.

**Results:**

Nine vessels in six patients with severe post-SAH vasospasm were stented. The stents were deployed in 16 vessel segments. All attempted implantations were technically successful. All patients demonstrated radiographic and clinical improvement of the vessel narrowing. No recurrent vasospasm or permanent vessel occlusion of the stented vessels was encountered. A thrombus formation in a Group 1 patient resolved under 4 mg eptifibatide IA infusion. During long-term angiographic follow-up, neither in-stent stenosis nor stent occlusion was found.

**Conclusions:**

Endovascular implantation of self-expanding stents is a potential *ultima ratio* strategy for patients with severe refractory post-SAH cerebral vasospasm. Stents with reduced thrombogenicity (avoiding DAPT) and bioabsorbable self-expanding stents might further advance this concept.

**Supplementary Information:**

The online version contains supplementary material available at 10.1186/s12883-022-02862-4.

## Introduction

Aneurysmal subarachnoid hemorrhage (aSAH) is a rare event that affects primarily young patients and therefore has a major social influence. Large-vessel vasospasm (i.e., vessel narrowing) had long been considered the sole or major cause of poor outcomes after aSAH. Recently, the focus of clinical research has shifted to other causes. Evidence suggests that patients with aSAH can develop DCI without angiographic vasospasm of large vessels [1–4]. Since 2004, when this phenomenon was first described by Kusaka [5], many studies have reported early brain injury. The sudden increase in intracranial pressure as a result of aSAH may lead to a decrease in cerebral blood flow [6] or to a breakdown in the blood–brain barrier and subsequent edema [7], thus leading to transient or persistent ischemia. Furthermore, several studies have reported the possible role of spreading depolarization after SAB in the development of cytotoxic, ionic, and vasogenic edema [8–11]. Distal cerebral vasospasm (CVS) after aSAH may also lead to microvascular dysfunction and microthrombosis [12]. Despite the above-mentioned reasons, CVS remains a leading cause of neurological deterioration after aSAH. CVS is responsible for a morbidity and mortality incidence of approximately 20% [13, 14]. Among patients with aSAH, 50–70% develop CVS, and approximately 30% present neurologic deficits [15, 16]. Despite limited accuracy, transcranial Doppler sonography (TCD) and transcranial color-coded Doppler (TCCD) remain preferred diagnostic methods in intensive care units for the detection of vasospasm, because of their logistic simplicity [17].

Computed tomography angiography (CTA) with computed tomography perfusion (CTP), and magnetic resonance angiography (MRA) are more reliable in CVS detection. Digital subtraction angiography (DSA) remains the gold standard for detecting and quantifying CVS [18]. Conservative management by intravenous or oral administration of calcium channel blockers has yielded sobering results [19]. Endovascular management strategies allow for effective treatment of CVS. These strategies include short-term intra-arterial administration of nimodipine [20], milrinone[21, 22], or verapamil [23]; long-term intra-arterial selective infusion of nimodipine [24]; and mechanical dilatation of large arteries with non-compliant balloons, compliant balloons [25–29], or stent-retrievers [30–34].

The substantial logistic efforts required for diagnosis and endovascular treatment outside the intensive care unit, and the complex cardiovascular and respiratory monitoring required before and during treatment, may lead to treatment delays and consequently poor outcomes. Despite the recent development of pharmacological and endovascular treatment options, the management of post-SAH CVS remains a challenge because of its frequent occurrence and the limited efficacy of the available treatment concepts.

Here, we report our anecdotal experience in the use of intracranial stenting for the treatment of patients with post-SAH CVS.

## Methods

### Study population

Between January 2010 and December 2021, we treated a total of 883 patients with aSAH.

The management strategy followed in our neurovascular center is as follows:

- Early diagnosis of SAH based on computed tomography (CT), CT angiography (CTA) or magnetic resonance imaging/angiography (MRI/MRA), followed by DSA.
- Early endovascular or microsurgical aneurysm treatment based on individualized multidisciplinary decision-making.
- Early external cerebrospinal fluid drainage when deemed necessary.
- Administration of nimodipine intravenous or per os starting on the first clinical day of stay, if tolerated.

During this period, 275 patients were diagnosed with post-SAH CVS. We retrospectively evaluated our database and identified 76 patients with *recurrent* CVS after initial short-term intra-arterial (IA) administration of milrinone (Corotrop, Sanofi-Avantis). These patients were treated with repeated IA administration of vasodilators and/or interventional mechanical vasodilatation of the vessel segments affected by CVS. The mechanical treatments applied were stent retriever-assisted angioplasty, pRELAX device-assisted angioplasty, and intracranial implantation of self-expandable stents. In six of these patients with CVS, intracranial self-expanding stent implantation was performed. Stent implantation was based on individual decision-making and was considered only in patients with severe and/or symptomatic vasospasm. A total of 70/76 (92.1%) patients with recurrent CVS remained without stent implantation. The clinical data files and the imaging results were available for retrospective evaluation. The patients’ demographic data and clinical characteristics are listed in Table 1.

**Table 1:** Demographic data and clinical characteristics of six patients with post-SAH vasospasm, treated with stent implantation

**Patient number**	Group	Age (years)	Sex	Initial HH	Fisher	Days between SAH and vasospasm onset	Days between SAH and stent implantation
1	1	61	F	1	3	0	0
2	1	48	F	3	2	0	0
3	2	52	F	3	4	4	15
4	2	49	F	5	4	7	8
5	2	41	M	5	4	6	7
6	2	67	F	3	4	5	6

### Data analysis

The clinical outcomes of the 70 patients with recurrent CVS who were not treated with stents were evaluated.

The case histories of the six stented patients were analyzed individually. Vasospasm was either prospectively diagnosed during the hospital stay or retrospectively identified by analysis of the imaging material by a neuroradiologist (with 3 years of experience in neuroradiological imaging), as supervised by the senior author. The six patients who underwent intracranial stenting were initially treated with IA vasodilators as soon as the vasospasms were angiographically diagnosed. After review of clinical data files and all imaging results (CT, MRI/MRA, and DSA) of the patients treated with intracranial stenting, the patients were divided into the following two groups according to the indication for stent implantation:

- Intracranial stenting during the endovascular therapy of a ruptured aneurysm, to allow for catheter access despite narrowing of the parent artery and in the context of stent-assisted coiling. In this group, the stents were required to treat the target aneurysm (Group 1).
- Intracranial stenting for recurrent vasospasm after failed IA infusion of vasodilators and/or mechanical treatment, particularly in patients with severe CVS who were at risk of brain infarction. In these patients, the stents were implanted independently of the treatment (Group 2).

### Follow-up

Early follow-up catheter angiography was performed before discharge according to routine clinical protocols. The first mid-term angiographic follow-up was performed 3–6 months after discharge. Clinical assessment was performed with the modified Rankin Scale (mRS) during the acute post-SAH period, at discharge, and in follow-up visits.

### Statistical analysis

For categorical variables, results are described as percentages. Continuous variables are reported as mean, median, and standard deviation.

## Results

### Patients with recurrent CVS managed without stenting

The 70 patients in the subgroup with recurrent CVS without stenting were treated with an individual combination of stellated ganglion blockade, IA short-term milrinone infusions, IA long-term nimodipine infusions, and mechanical treatment. The clinical outcomes of this subgroup, assessed at discharge and at 6 months, respectively, indicated the following: mRS 0–2 in 25/70, 46/70 patients (35.7%, 65.7%), mRS 3–5 in 39/70, 18/70 patients (55.7%, 25.7%), and mRS 6 in 6/70, 6/70 patients (8.6%, 8.6%).

#### Group 1

This group consisted of two patients and represented our initial experience in intracranial stenting as a treatment for recurrent CVS. Both patients showed CVS on day 1 during the first DSA examination to locate the source of hemorrhage. At that time, we suspected that the current SAH was not the first event. This suspicion was confirmed in one patient, according to information provided by the family regarding a severe headache 7 days earlier. Both patients showed neurological deficits due to CVS. Therefore, endovascular treatment could not be delayed. Endovascular coil occlusion of the ruptured aneurysm was planned for both patients. Initial treatment with IA vasodilators was performed in both patients for approximately 30 min immediately before the start of endovascular coiling of the ruptured aneurysm. After an infusion of 8 mg milirinone into the internal carotid artery (ICA), the affected vessel segments showed sufficient vasodilation to enable coil occlusion of the aneurysms. During the endovascular coiling of the ruptured aneurysms, recurrent vasospasm was detected in both patients. Concurrently the implanted coils shifted into the parent vessels. Vasospasm and coil displacement resulted in a substantial perfusion delay of the dependent vasculature.

To be able to continue the treatment, prevent the increase in CVS, and prevent ischemic damage, we implanted a self-expanding stent into the parent artery. Consequently, the covered vessel segments dilated, the perfusion of the distal vessels improved, and we completed the treatment. The post-procedural CT imaging showed no ischemic lesions due to CVS. On day 1 after aneurysm coiling, one patient showed generalized moderate vasospasm in the proximal segments of the middle and anterior cerebral artery, excluding the stented M1-M2 vessel segments. In both patients, the injection of IA vasodilators for 30 min induced significant dilatation of the affected vessels and vessel segments. After that, the patients showed no further recurrent vasospasm, ischemic lesions, or new neurological deficits during the remainder of their clinical stay.

#### Group 2

This group included four patients with a median age of 50.5 years (IQR 58.7- 48.2). Three of these patients were in a poor clinical condition (two patients with an initial HH V score, one with secondary deterioration), and the clinical management was complicated by peri- and postprocedural issues.

One patient was diagnosed with a periprocedural dissection of the left ICA after endovascular treatment of a giant left ICA aneurysm with stent-assisted coiling (pCONUS, phenox GmbH). This dissection caused an ischemic lesion 6 days later by increasing vasoconstriction due to vasospasm. Another patient was found to have an intracerebral hemorrhage in the brain’s left frontal lobe after a new external ventricular drain was inserted 5 days after endovascular treatment of a giant aneurysm of the right ICA with flow diverter-assisted coiling. The third patient showed a new postoperative hemorrhage on non-contrast CT performed immediately after microsurgical clipping of an aneurysm of the right middle cerebral artery (MCA) bifurcation.

Beyond the recurrent generalized CVS, all these conditions increased the intracranial pressure in the patients and complicated their clinical management. One patient underwent hemicraniectomy as a result of the rebleeding. The fourth patient experienced only headaches at the beginning of the clinical stay. One day later, the patient showed rapid high-grade neurological deficits due to CVS. All patients in this group showed recurrent CVS and therefore underwent at least one independent therapy session before stent implantation and multiple endovascular procedures (median 3; 2–3).

Except for the two patients in group 1, intracranial stenting as a treatment for CVS was not performed in the first vasospasm treatment session. Three patients also underwent interventional mechanical treatment with a stent retriever or pRELAX device (femtos GmbH). In two patients, we performed IA continuous selective infusion of nimodipine for 3–6 days because of generalized CVS in other vascular supply regions. At the beginning of our experience, we used intracranial stenting in CVS to dilate the most affected or relevant vessel segments by using only one stent. Later, with increasing experience, we have decided to insert as many as four stents in a patient with generalized recurrent CVS. The concerning vessel segments in these patients were the ICA bifurcation, A1, A2, AcomA, M1, and M2. We performed stenting of two to five vessel segments on each patient. A total of 18 artery segments were covered with stents. Ten stents were used, and we successfully implanted the stents into the spastic segments after IA vasodilator infusion in all cases. The medications and interventional characteristics are listed in Table 2. After the stenting procedures, all vessel segments showed satisfactory vasodilation and improved perfusion of the distal vasculature. The IV administration of nimodipine was continued for 14, 7, 9, 19, 11, and 8 days. Three patients required further endovascular CVS treatment (patient 2: short-term intra-arterial administration of nimodipine, owing to vasospasm in the ACA; patient 4: intra-arterial selective infusion of nimodipine for 9 days with microcatheters, owing to general vasospasm, with the exception of the segments treated with stent implantation and the distal vessels thereof; patient 5: short-term intra-arterial administration of nimodipine, owing to vasospasm in the left MCA and ACA, followed by mechanical balloon dilatation of the left ACA because of persistent high-grade vasospasm), but not for the stented vessel segments. No evidence of any luminal narrowing of the stented vessels was observed. No thromboembolic or spasm-related ischemic lesions were detectable in the supply area of the treated vessels in follow-up CT or MRI after stent implantation. Most patients (five of six, 83.3%) showed neurological improvement at discharge, and all patients showed neurological improvement at the 6-month follow-up. A good clinical outcome (mRS ≤ 2) was achieved in five of six patients (Table 3).

**Table 2:** Stenting as bail-out treatment of vasospasm after aSAH: loading medication, stent dimensions, target vessel, vessel segment, Nimodipine IV after stenting, thrombus formation, bleeding complication

Patient number	Group	Loading medication	Stent dimensions	Target vessel	Vessel segments (diameters in mm)	Nimodipine IV after stenting (days)	Thrombus after stenting	Peri- or postprocedural bleeding
1	1	1 × 500 mg ASA 1 × 75 mg PRA	Solitaire 4/15	ACA rt	A1-A2 (0.7–0.5 mm)	Yes (14 days)	no	no
2	1	1 × 4 mg EPT 2 × 500 mg ASA 1 × 600 mg CLO	LVIS Jr 2.5/24	MCA rt	ICA-M1-M2 (1–0.7–0.4 mm)	Yes (7 days)	Transient Thrombus formation resolved: EPT IA	no
3	2	1 × 500 mg ASA 1 × 30 mg PRA	Enterprise 4/39	MCA rt	M1-M2 (0.4–0.5 mm)	Yes (9 days)	no	no
4	2	1 × 12.4 mg EPT 1 × 500 mg ASA 1 × 180 mg TIC	Enterprise2 4/30	MCA rt	M1-M2 (0.3–0.7 mm)	Yes (19 days)	no	no
5	2	1 × 500 mg ASA 1 × 10 mg PRA	Enterprise2 4/16 1 × Enterprise2 4/23	MCA rt ACA rt	M1 (0.7 mm) A1-A2 (0.6–0.7 mm)	Yes (11 days)	no	no
6	2	1 × 12.4 mg EPT 1 × 500 mg ASA 1 × 180 mg TIC	Neuroform Atlas 3/15 Baby Leo 2/12 Neuroform Atlas 4/24 Neuroform Atlas 4.5/30	ACA rt ACA rt MCA lt ICA lt	A2 (0.5 mm) A1 (0.6 mm) ICA-M1 (1.7–0.6 mm) A1-A2 (0.5–0.4 mm)	Yes (8 days)	no	no

**Table 3:** Stenting as bail-out treatment of vasospasm after aSAH. MFV before and 1 day after stenting; mRS before and after stenting

Patient number	Group	MFV before stenting	mRS before stenting	MFV at day 1 after the stenting	mRS at discharge	mRS 90 days	mRS 180 days	Last mRS (days)
1	1	_ MCA	2	50	1	0	0	0 (3320)
2	1	160 MCA	5	90	3	2	0	0 (1984)
3	2	250 MCA	5	100	4	3	3	3 (905)
4	2	240 MCA	5	95	4	3	2	2 (1080)
5	2	180 MCA	5	80	3	2	2	2 (282)
6	2	160 MCA	4	100	0	0	0	0 (466)

Group 1: intracranial stenting of refractory CVS during endovascular treatment of the ruptured aneurysm. Group 2: intracranial stenting of refractory CVS after failure of IA vasodilators or mechanical treatment, independently of aneurysm treatment. TCD before stenting: values in cm/sec, with the relevant vessels; MCA: middle cerebral artery, ACA: anterior cerebral artery

### Complications

One technical complication occurred. In the first group, patient 2 showed a thrombotic occlusion of the stented MCA. The thrombus resolved with the administration of eptifibatide IA (Integrilin, GlaxoSmithKline). This complication did not lead to clinical consequences. The patient showed no associated ischemic lesions after the procedure. No complications occurred in the remaining patients. No peri- or post-procedural hemorrhage or vascular injuries were encountered.

## Discussion

Although the endovascular and microsurgical treatment of ruptured aneurysms has substantially improved during the past three decades, similar progress in the treatment of CVS is lacking. The past 20 years have seen a mindset shift from a fatalistic acceptance of poor outcomes due to CVS toward active attempts to address these issues. The search for a safe and efficacious treatment for CVS has been ongoing since the 1970s [35]. Jabbar et al. [36] have compared two cohorts including 1057 patients. All patients underwent daily TCD ultrasonography to detect CVS. Patients in group A were treated immediately after any suspicion of CVS, regardless of the TCD results. Patients in group B were treated by endovascular means only after persistent CVS despite induced hypertension for 2–4 h or when a mean flow velocity above 160 cm/sec was gradually exceeded for two consecutive days. In group A, 24.4% of patients underwent the first endovascular treatment on day 6 ± 3.64, and in group B, 14.4% underwent the first endovascular treatment on day 8.9 ± 4.78. The rate of DCI was lower in cohort A (20.8% vs. 29%, *p* = 0.0023, OR 0.64, 95% CI 0.48–0.85), as confirmed by multivariate analysis (*p* = 0.001, adjusted OR 0.59, 95% CI 0.44–0.8). The rates of DCI were higher in patients undergoing endovascular treatment for delayed ischemic neurologic deficit than in those undergoing endovascular treatment solely because of TCD measurements (64% vs. 44.7%, *p* = 0.0277). The rate of unfavorable outcomes after SAH was also lower in cohort A (44% vs. 56%, *p* = 0.0404. This study demonstrated that early identification and aggressive treatment might result in better functional outcomes.

Balloon dilatation of proximal arteries (mainly the distal ICA and the proximal MCA) is an acceptable option, but the risk of vessel dissection and even rupture remains a concern.

In recent years, endovascular mechanical vessel dilatation using stent retrievers (e.g., Solitaire, Medtronic; pRESET, phenox) has been established.

The non-selective IA injection of vasodilators can lead to an steal phenomenon due to a significantly increased inflow in the non-spastic arteries [37]. To avoid this steal phenomenon, distal vasospasm was suggested to be best treated with medication after initial mechanical treatment of the proximal vasospasm. Balloon angioplasty [13–17] and “stentoplasty” [18–22] have shown therapeutic efficacy and sustained improvement. Although balloon angioplasty was initially believed to damage the extracellular matrix, evidence has indicated that balloon angioplasty of an arterial segment instead induces paralysis of the vessel without necessarily damaging the underlying extracellular matrix [32, 38]. The induced vessel paralysis after angioplasty is affected by the contractile state of the vessel. Contracted (vasospastic) vessels, require less dilatation to become paralyzed. Therefore, vessels with CVS are predisposed to paralysis after mechanical dilation and thus can be treated with devices with lower radial force [32]. Damage to the underlying smooth muscle cells, which stiffen when contracted and may potentially be more prone to mechanical disruption, has been suggested to explain this phenomenon [39]. This finding suggests that mechanical angioplasty should be performed before chemical angioplasty. Simultaneously, lower forces might be required, thus potentially explaining why stent retrievers have shown some success in treating CVS despite having much lower radial force than balloons. Kwon et al. [32] compared patients in whom the stent was deployed initially followed by injection of a vasodilator (nicardipine) and those in whom a vasodilator was injected first, and stentoplasty was performed second. In the vasodilator-first group, 71.4% of treated vessel segments (10/14) showed vasodilation after stentoplasty, but 60% of patients (3/5) developed recurrent vasospasm requiring repeated angioplasty. In the stentriever-first group, 82.1% of segments (32/39) showed vasodilatation after stentoplasty, but none of the patients developed radiological or clinical evidence of recurrent vasospasm. This small clinical study corroborated the initial hypothesis suggested by Bhogal et al. [33], building on the work of Fischell et al. [38, 39].

Bhogal et al. [33] have noted the following benefits of treating CVS with recheatable stent retrievers, as specifically compared with vasodilator medications or balloon angioplasty:

- No flow arrest
- Ability to track stents into the distal vessel segments
- Familiarity with the use of stents from mechanical thrombectomy
- A likely lower risk of perforation because of operator-independent stent expansion
- Long-lasting and durable vasodilation
- Short procedure time
- Ability to inject vasodilating drugs at the same time

Similarly, Su et al. [34] have evaluated the safety and efficacy of treatment of CVS with stent retriever-assisted angioplasty. In this study, 14 vessels from six patients with resistant CVS showed improvements in vessel diameter, and all patients had improved neurological examination findings.

Intracranial stenting offers the same above-mentioned advantages because of the similar release concept of the two devices in the distal vessels. In extremely resistant CVS cases, intracranial stenting offers a continuous effect as a bail-out option, mainly because of the permanent dilatation of the stented vessels and the better circulation of the vasodilating drugs in the affected distal segments that cannot be reached by angioplasty.

Stent implantation has also been reported. Andic et al. [40] have analyzed data from 15 consecutive patients with 18 aneurysms, eight of whom underwent stent-assisted coiling. In most cases (*n* = 6), an LVIS Jr. (MicoVention) stent was implanted, with a Solitaire (Medtronic) or Acclino (Acandis) stent used in the remainder. The authors observed moderate to complete dilatation of the spastic parent arteries after deployment of the stents in patients treated with stent-assisted coiling. In one case, refractory vasospasm occurred after the treatment of two aneurysms and the authors found no evidence of recurrent vasospasm in the stented segments but observed widespread recurrent vasospasm in the segments previously treated with chemical angioplasty.

Although these findings were based on only a small series, this article highlights the potential for braided and laser-cut stents to effectively treat and prevent recurrent CVS. Subsequently, Bhambri et al. [41] described a novel method of using drug-eluting stents to treat CVS. The authors developed polymer-coated laser-cut stents and used various methods to coat the stent. The laser-cut and polymer-coated stents were also loaded with different doses of verapamil with varying drug release pharmacokinetics. In all cases, the combined stent demonstrated an initial burst phase of drug release followed by sustained drug release. Varying the concentration of the verapamil changed these different phases by altering the construction of the polymer coating. This preliminary in vitro study suggests the potential promise of further developing dedicated stents for vasospasm that release vasodilating drugs.

Despite the development of endovascular techniques and their demonstrated effectiveness in treating recurrent vasospasm, some of these therapies remain unsuccessful. The possible reasons for the failure of endovascular treatment are as follows:

- Steal phenomena, particularly with intra-arterial vasodilator treatment
- Distal location: difficult navigation and dilatation of CVS in distal vessel segments for endovascular mechanical treatments
- Tortuosity and curved vessels: complex dilatation of spastic vessels in a curved course, such as the transition from the A1 to the A2 segment, particularly for balloon angioplasty

Some endovascular treatments have a short-term effect, but CVS is a dynamic and potentially long-lasting pathology during the first 3 weeks after aSAH. Recurrent CVS may not be successfully treated in some patients despite multiple endovascular treatments sessions. Consequently, the search for a therapeutic solution for refractory CVS continues. Intracranial stenting of vessels with vasospasm is an *ultima ratio* treatment in patients with severe recurrent vasospasm. We have attempted to enforce permanent dilatation of vessels with resistant vasospasms by stent implantation. The implanted device is intended to avoid any steal phenomenon and enhance vasodilator drugs’ effects, particularly on the peripheral vessels. By improving perfusion in the proximal vessels, we have also observed a reduction of CVS in the distal vessels.

This level of improvement occurred only after stent implantation and can therefore not be explained by the previous IA administration of the vasodilator.

The concept of treating CVS with intracranial stenting arose from incidental experience during the management of complications from aneurysm coil occlusion. During coiling, impaired perfusion of the parent vessel and the dependent vasculature was observed, owing to inadvertent displacement of the implanted coils. Stents were implanted to maintain the coils inside the aneurysm and restore normal blood flow. The vessels where the stents were implanted had shown CVS before and during the coiling despite the IA vasodilator administration. After stenting, we observed decreased vasospasms with similar perfusion improvement in the distal supply territory. One of the patients was diagnosed with high-grade CVS after day 1, but not within or distal to the stented vessel. After our initial two incidental experiences and the excellent long-term outcome, we decided to perform this treatment in the four described cases after several unsuccessful chemical and mechanical treatments. In the first two patients, we implanted only one stent in the MCA in the M1-M2 junction. With experience, in the third patient, we implanted two stents in the middle and anterior cerebral artery ipsilaterally. In the last patient, four stents were implanted, two on each side. The stent implantations were always performed under dual antiplatelet therapy (DAPT). Overall, only one transient thromboembolic occlusion occurred as a periprocedural complication. None of the patients showed postprocedural hemorrhage, in-stent stenosis, or DCI of the entire vascular territories of the stented vessels. DAPT was maintained in patients for 6 months. Antiplatelet therapy was de-escalated to single antiplatelet therapy after the 6-month DSA follow-up.

The repetition of endovascular treatment of recurrent vasospasm increases the risk of periprocedural complications and radiation exposure [42]. Endovascular treatment may not always be feasible, owing to the complicated clinical management of patients with aSAH, particularly respiratory and cardiovascular management. Nonetheless, we believe that it is a viable treatment option as a last resort in refractory vasospasm to avoid life-threatening DCI after the failure of medicinal and mechanical treatments.

The majority of neurovascular stents (e.g., Enterprise, Cerenovus; Solitaire, Medtronic; LVIS, MicroVention) require microcatheters with an 0.021″ ID. These stents have a significantly higher radial force than their counterparts with a reduced crossing profile. Neuroform Atlas (Stryker), LVIS Jr (MicroVention), and pEGASUS (phenox) can be implanted through 0.017″ ID microcatheters. Three of the implanted stents were Neuroform Atlas. The low profile 0.017″ ID microcatheter (e.g., Excelsior SL-10, Stryker) allows for atraumatic navigation of distal and narrow vessel segments. The relatively low radial force was not an issue. 

### Limitations

The technique of stenting as a treatment for CVS and the present study have several limitations.

The currently available stents are not designed for this purpose, and their use for this indication is off-label. Technical specifications such as radial force, cell structure (open versus closed cell), and cell dimensions have not been evaluated. Space certainly exists for an optimized implant. All stents that we have used to date were uncoated and require DAPT. The need for DAPT during the acute phase after aSAH is a matter of concern. pEGASUS (femtos) has a hydrophilic coating and can be implanted under SAPT. Patients in the acute phase after aSAH have elevated platelet activity and usually require increased dosages of antiplatelet medication for the intended platelet function inhibition.

The data presented herein come from anecdotal cases treated under emergency circumstances according to the knowledge and experience of the senior author. The available devices for the mechanical treatment of CVS (e.g., pRELAX, femtos) have a CE mark for this indication. A large scale randomized controlled trial is in preparation in Germany. In this trial, stenting may become an option if the temporary deployment of pRELAX does not prevent the recurrence of vasospasm. Stenting proximal arteries is only one option in the armamentarium for the treatment of post-SAH vasospasm.

## Conclusions

The ruptured aneurysm should be secured first. Implantation of self-expanding stents, although not commonly used for the treatment of post-SAH recurrent CVS, is very effective acutely and in the long term, and is safe if the antiplatelet medication is tested and adjusted accordingly. This therapy is considered an *ultima ratio* after the failure of standard pharmacological and interventional measures. The treatment of CVS might be one of the few reasonable neurovascular indications for bioabsorbable stents.

## Supplementary Information

**Additional file 1.**

## Data Availability

The anonymized but otherwise complete datasets generated during the current study are available as supplementary material with this publication.

## Abbreviations

AcomA: Anterior communicating artery
ASA: Acetylsalicylic acid
aSAH: Aneurysmal subarachnoid hemorrhage
CLO: Clopidogrel
CT: Computed tomography
CTA: Computed tomography angiography
CTP: Computed tomography perfusion
CVS: Cerebral vasospasm
DAPT: Dual antiplatelet therapy
DCI: Delayed cerebral ischemia
DSA: Digital subtraction angiography
EPT: Eptifibatide
HH: Hunt and Hess
IA: Intra-arterial
ICA: Internal carotid artery
ID: Inner diameter
MCA: Middle cerebral artery
MRA: Magnetic resonance angiography
MRI: Magnetic resonance imaging
mRS: Modified Rankin Scale
MFV: Mean flow velocity
PRA: Prasugrel
SAH: Subarachnoid hemorrhage
TCCD: Transcranial color-coded doppler sonography
TCD: Transcranial Doppler sonography
TIC: Ticagrelor
