# Supplementary material for: Intracranial stenting as a bail-out option for posthemorrhagic cerebral vasospasm: a single-center experience with long-term follow-up
Source: BMC Neurol. 2022 Sep 15;22:351. doi: 10.1186/s12883-022-02862-4 (PMC9476569; doi:10.1186/s12883-022-02862-4)
Supplement: Supplementary file 1 — Additional file 1. [file 12883_2022_2862_MOESM1_ESM.pptx]

## Slide 1
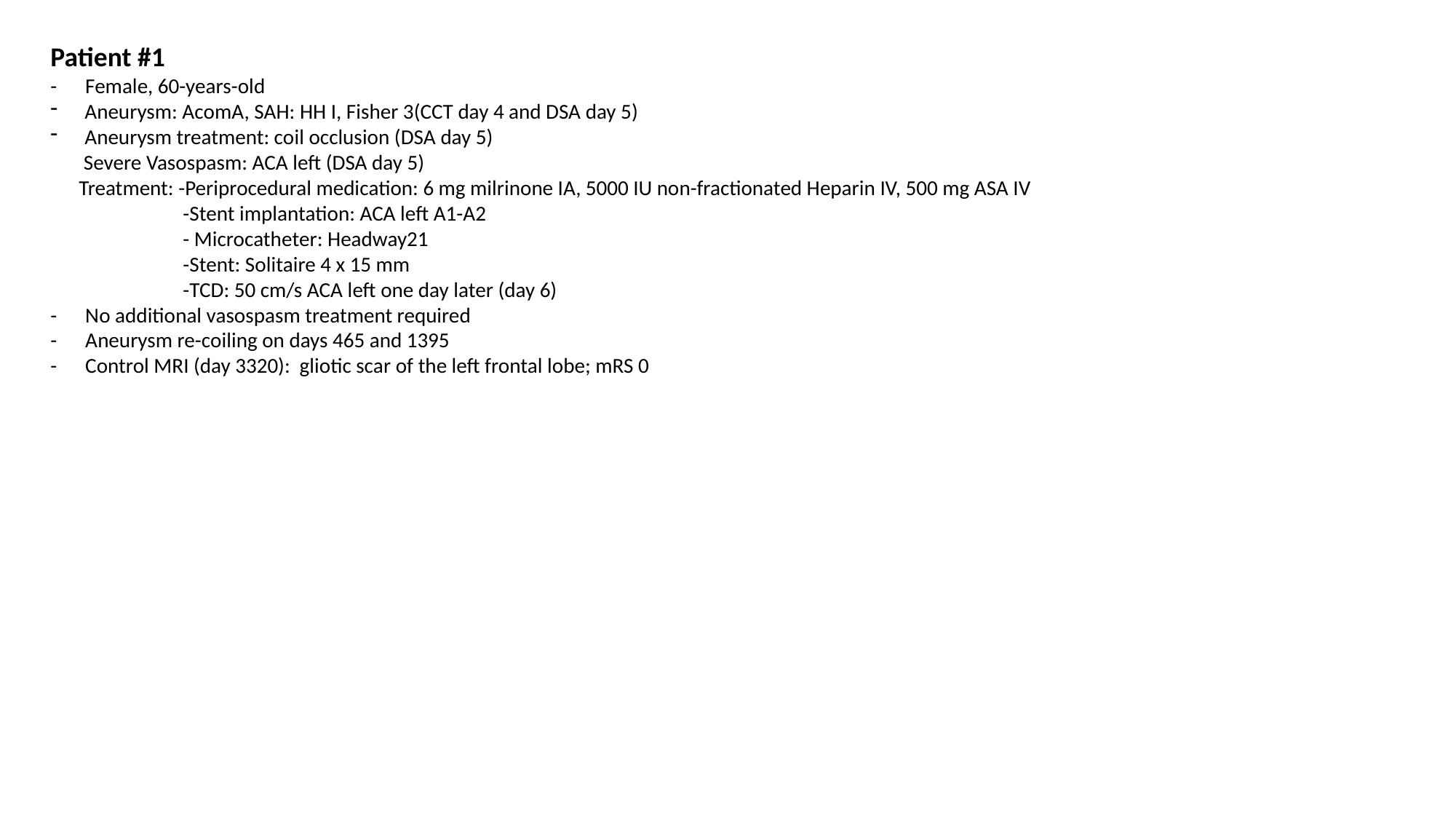

Patient #1
- Female, 60-years-old
Aneurysm: AcomA, SAH: HH I, Fisher 3(CCT day 4 and DSA day 5)
Aneurysm treatment: coil occlusion (DSA day 5)
 Severe Vasospasm: ACA left (DSA day 5)
 Treatment: -Periprocedural medication: 6 mg milrinone IA, 5000 IU non-fractionated Heparin IV, 500 mg ASA IV
 -Stent implantation: ACA left A1-A2
 - Microcatheter: Headway21
 -Stent: Solitaire 4 x 15 mm
 -TCD: 50 cm/s ACA left one day later (day 6)
- No additional vasospasm treatment required
- Aneurysm re-coiling on days 465 and 1395
- Control MRI (day 3320): gliotic scar of the left frontal lobe; mRS 0

## Slide 2
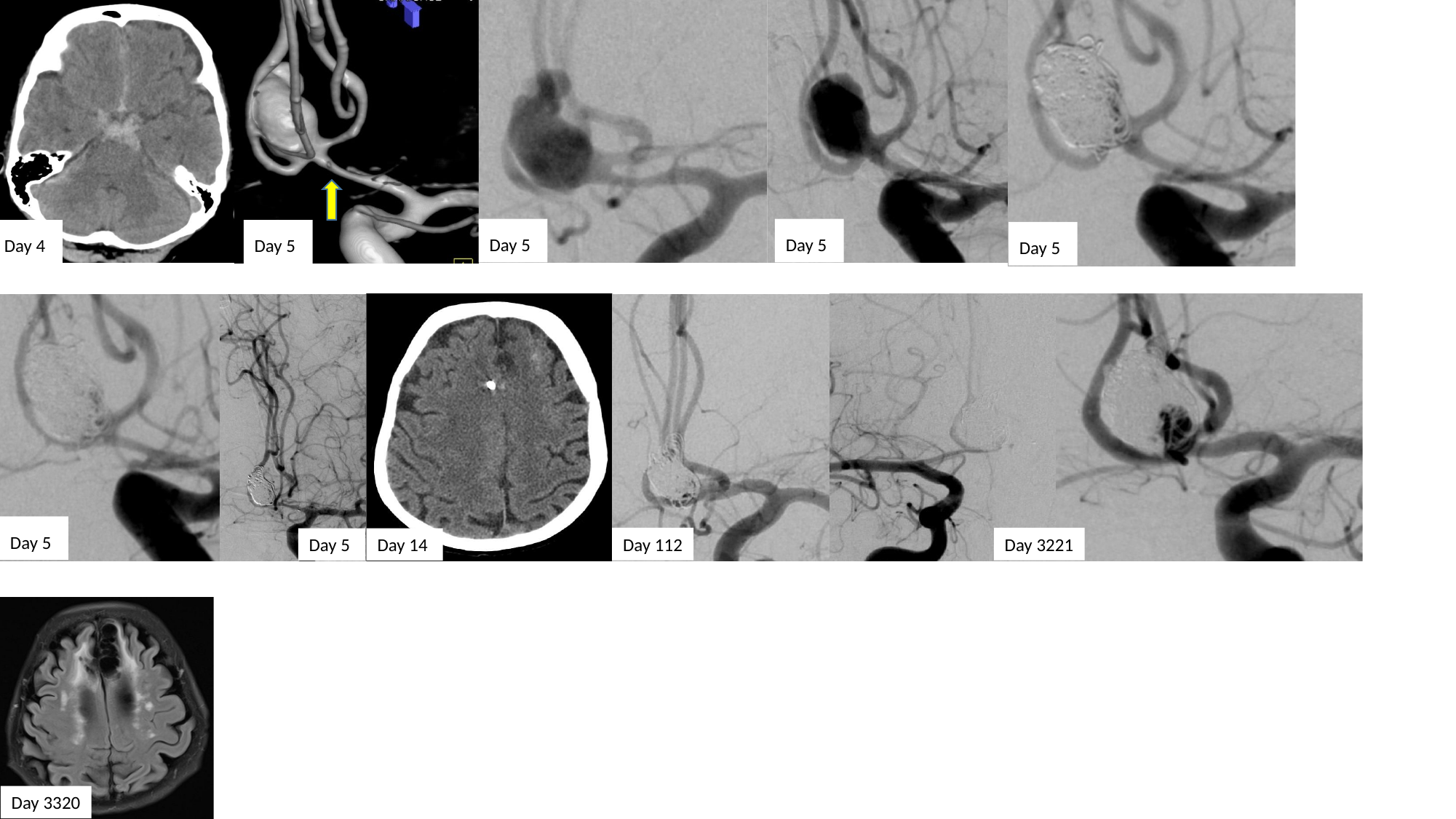

Day 5
Day 5
Day 4
Day 5
Day 5
Day 5
Day 112
Day 3221
Day 5
Day 14
Day 3320

## Slide 3
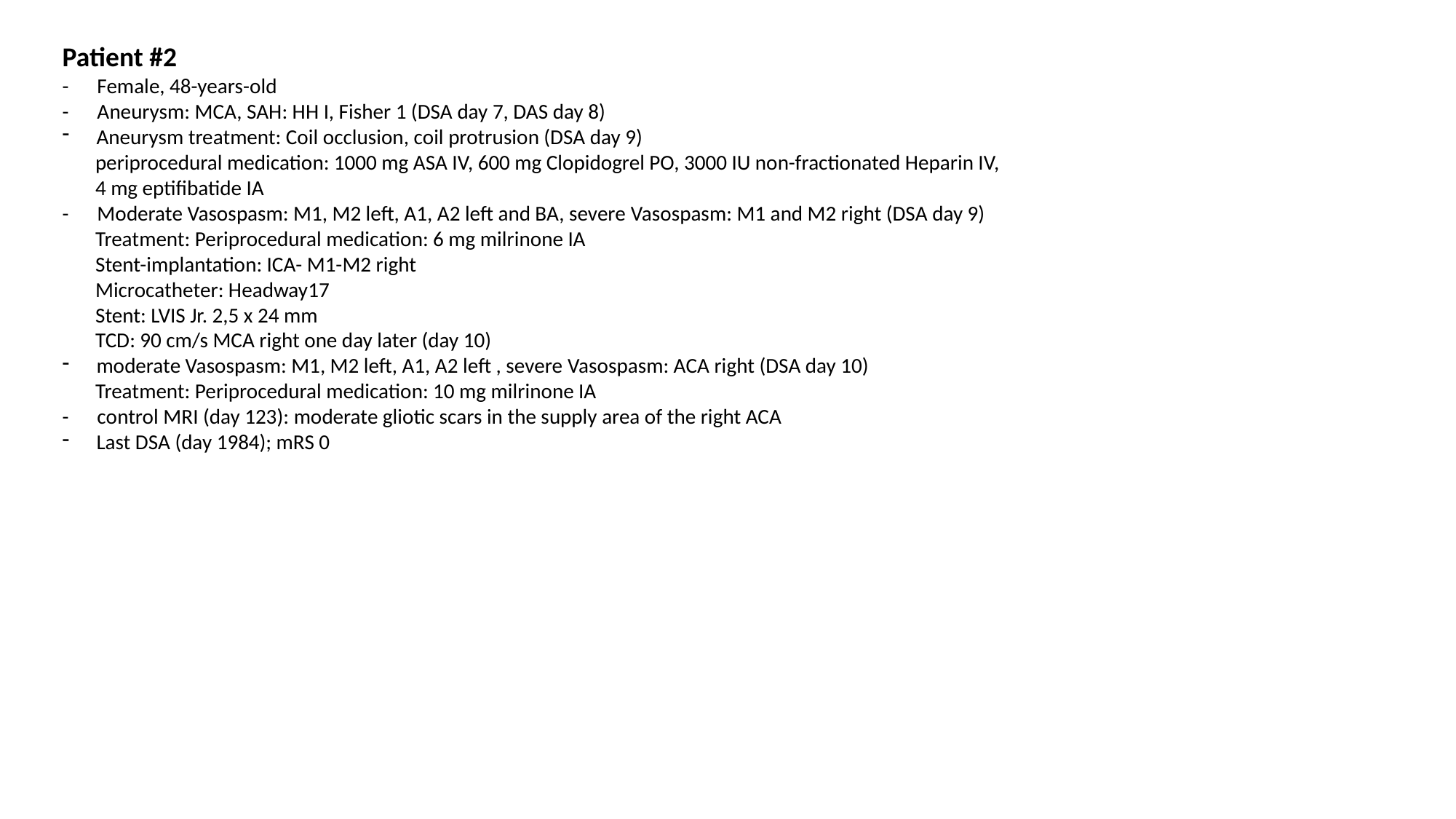

Patient #2
- Female, 48-years-old
- Aneurysm: MCA, SAH: HH I, Fisher 1 (DSA day 7, DAS day 8)
Aneurysm treatment: Coil occlusion, coil protrusion (DSA day 9)
 periprocedural medication: 1000 mg ASA IV, 600 mg Clopidogrel PO, 3000 IU non-fractionated Heparin IV,
 4 mg eptifibatide IA
- Moderate Vasospasm: M1, M2 left, A1, A2 left and BA, severe Vasospasm: M1 and M2 right (DSA day 9)
 Treatment: Periprocedural medication: 6 mg milrinone IA
 Stent-implantation: ICA- M1-M2 right
 Microcatheter: Headway17
 Stent: LVIS Jr. 2,5 x 24 mm
 TCD: 90 cm/s MCA right one day later (day 10)
moderate Vasospasm: M1, M2 left, A1, A2 left , severe Vasospasm: ACA right (DSA day 10)
 Treatment: Periprocedural medication: 10 mg milrinone IA
- control MRI (day 123): moderate gliotic scars in the supply area of the right ACA
Last DSA (day 1984); mRS 0

## Slide 4
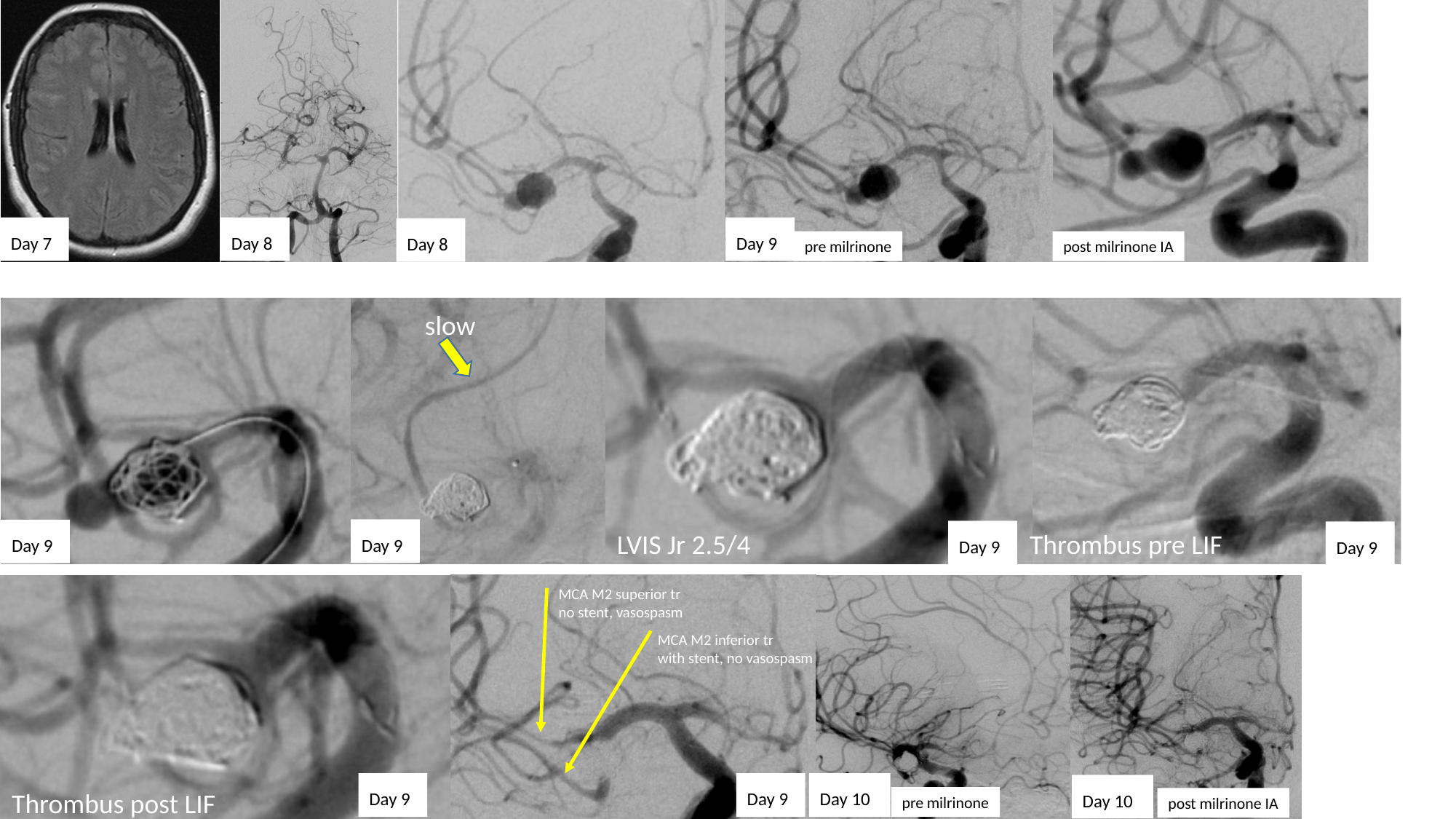

Day 7
Day 8
Day 9
Day 8
pre milrinone
post milrinone IA
slow
Day 9
Day 9
Day 9
Day 9
LVIS Jr 2.5/4
Thrombus pre LIF
MCA M2 superior tr
no stent, vasospasm
MCA M2 inferior tr
with stent, no vasospasm
Day 9
Day 9
Day 10
Day 10
Thrombus post LIF
pre milrinone
post milrinone IA

## Slide 5
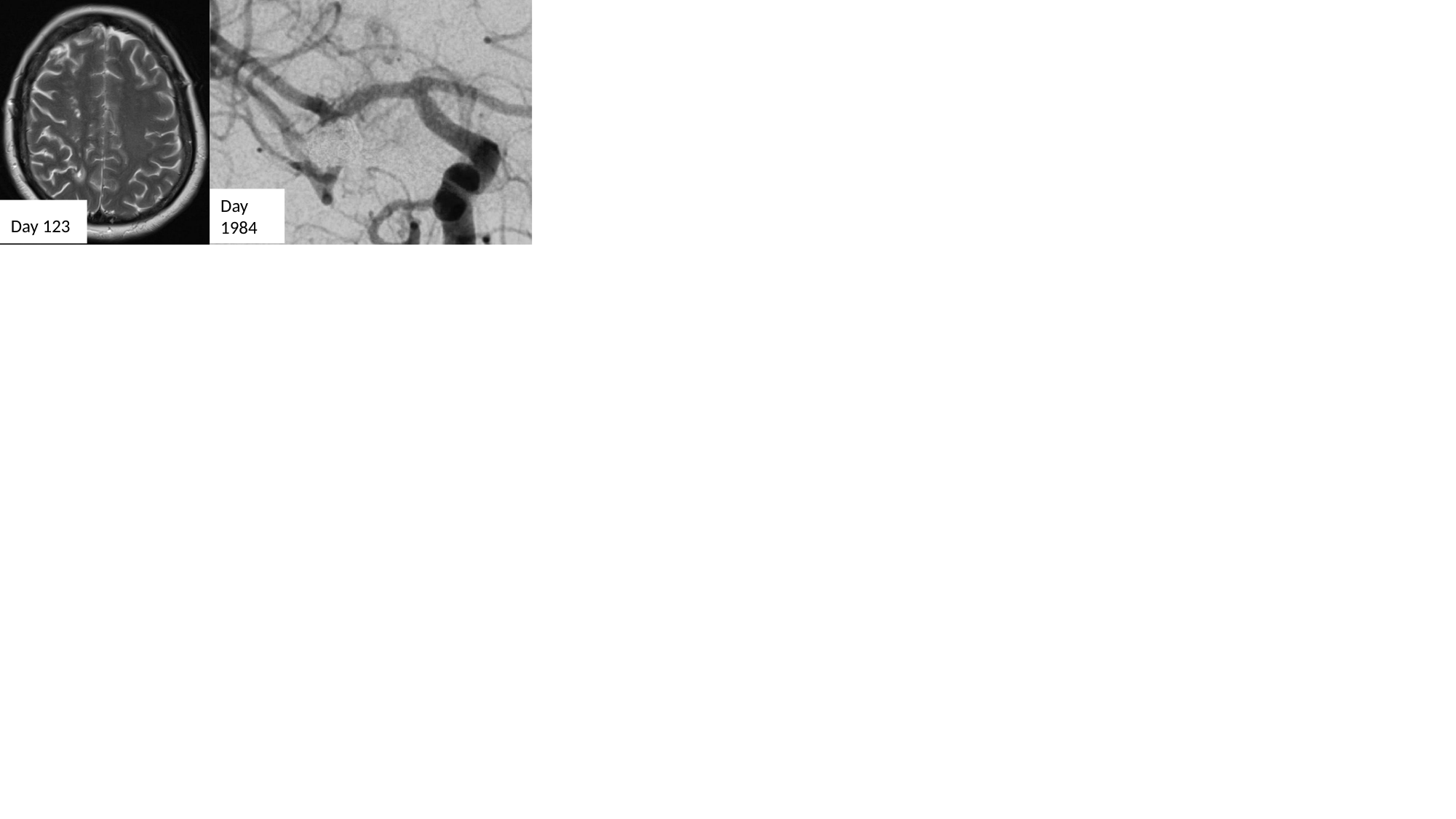

Day 1984
Day 123

## Slide 6
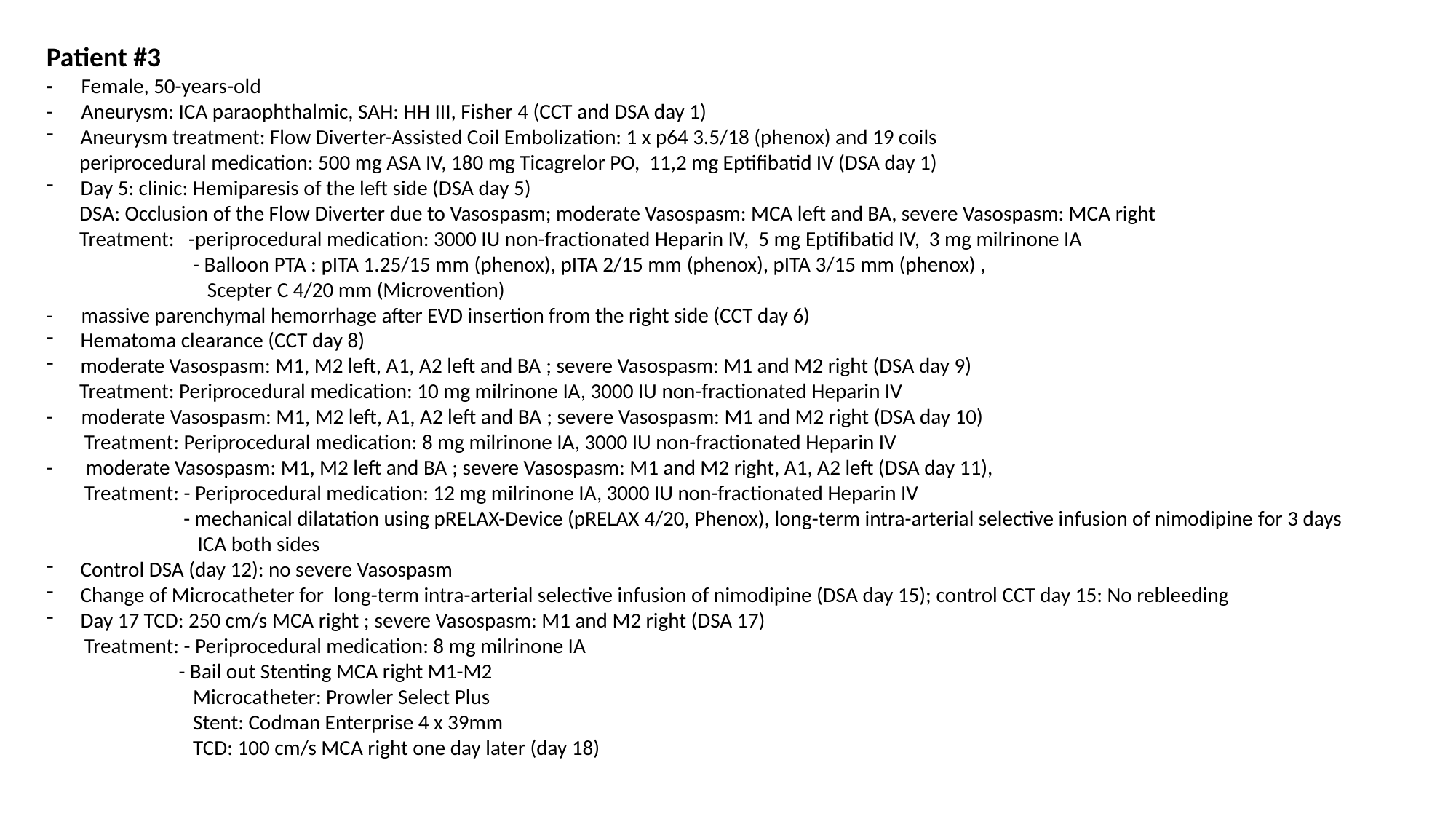

Patient #3
- Female, 50-years-old
- Aneurysm: ICA paraophthalmic, SAH: HH III, Fisher 4 (CCT and DSA day 1)
Aneurysm treatment: Flow Diverter-Assisted Coil Embolization: 1 x p64 3.5/18 (phenox) and 19 coils
 periprocedural medication: 500 mg ASA IV, 180 mg Ticagrelor PO, 11,2 mg Eptifibatid IV (DSA day 1)
Day 5: clinic: Hemiparesis of the left side (DSA day 5)
 DSA: Occlusion of the Flow Diverter due to Vasospasm; moderate Vasospasm: MCA left and BA, severe Vasospasm: MCA right
 Treatment: -periprocedural medication: 3000 IU non-fractionated Heparin IV, 5 mg Eptifibatid IV, 3 mg milrinone IA
 - Balloon PTA : pITA 1.25/15 mm (phenox), pITA 2/15 mm (phenox), pITA 3/15 mm (phenox) ,
 Scepter C 4/20 mm (Microvention)
- massive parenchymal hemorrhage after EVD insertion from the right side (CCT day 6)
Hematoma clearance (CCT day 8)
moderate Vasospasm: M1, M2 left, A1, A2 left and BA ; severe Vasospasm: M1 and M2 right (DSA day 9)
 Treatment: Periprocedural medication: 10 mg milrinone IA, 3000 IU non-fractionated Heparin IV
- moderate Vasospasm: M1, M2 left, A1, A2 left and BA ; severe Vasospasm: M1 and M2 right (DSA day 10)
 Treatment: Periprocedural medication: 8 mg milrinone IA, 3000 IU non-fractionated Heparin IV
- moderate Vasospasm: M1, M2 left and BA ; severe Vasospasm: M1 and M2 right, A1, A2 left (DSA day 11),
 Treatment: - Periprocedural medication: 12 mg milrinone IA, 3000 IU non-fractionated Heparin IV
 - mechanical dilatation using pRELAX-Device (pRELAX 4/20, Phenox), long-term intra-arterial selective infusion of nimodipine for 3 days
 ICA both sides
Control DSA (day 12): no severe Vasospasm
Change of Microcatheter for long-term intra-arterial selective infusion of nimodipine (DSA day 15); control CCT day 15: No rebleeding
Day 17 TCD: 250 cm/s MCA right ; severe Vasospasm: M1 and M2 right (DSA 17)
 Treatment: - Periprocedural medication: 8 mg milrinone IA
 - Bail out Stenting MCA right M1-M2
 Microcatheter: Prowler Select Plus
 Stent: Codman Enterprise 4 x 39mm
 TCD: 100 cm/s MCA right one day later (day 18)

## Slide 7
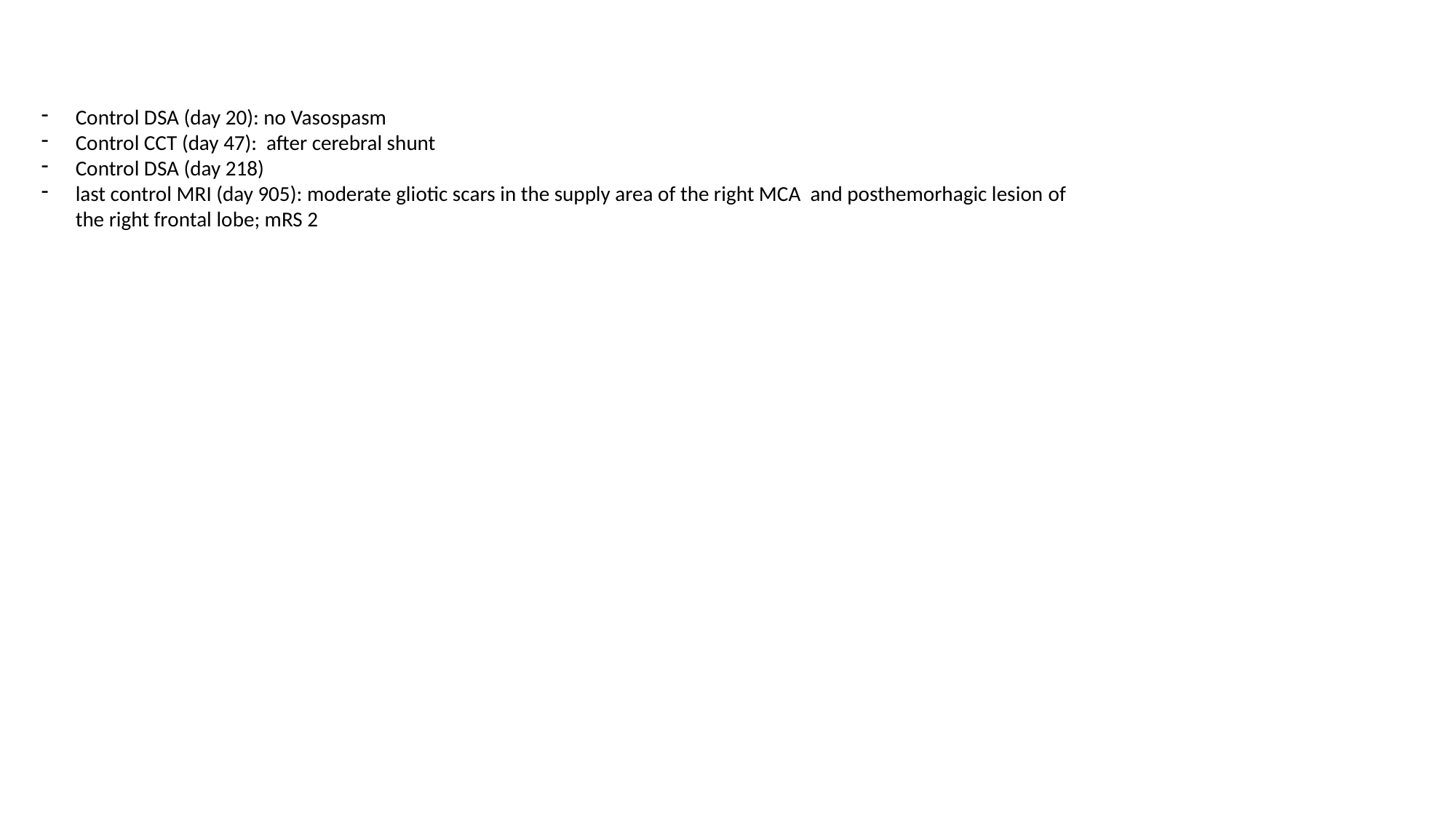

Control DSA (day 20): no Vasospasm
Control CCT (day 47): after cerebral shunt
Control DSA (day 218)
last control MRI (day 905): moderate gliotic scars in the supply area of the right MCA and posthemorhagic lesion of the right frontal lobe; mRS 2

## Slide 8
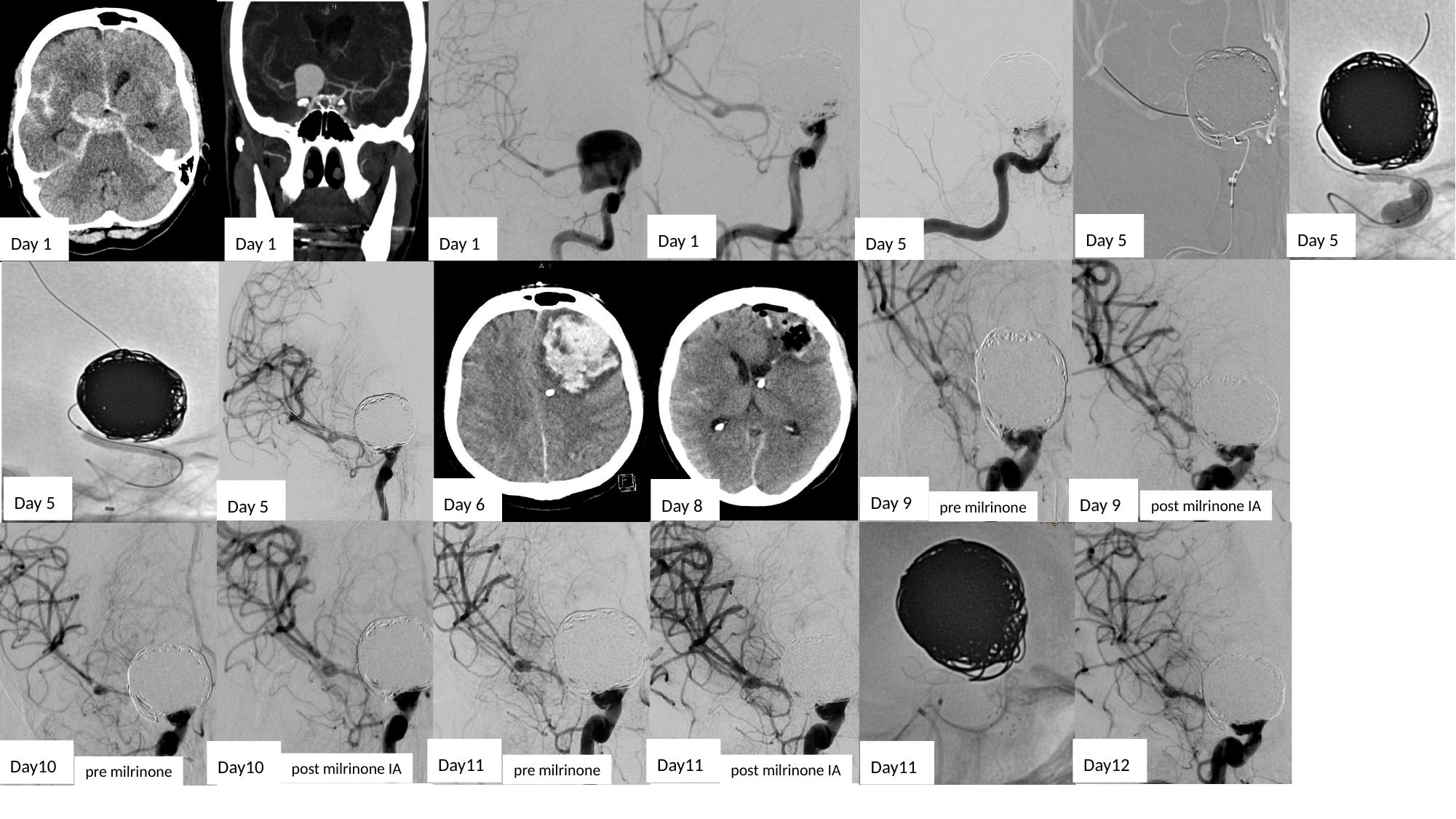

Day 5
Day 5
Day 1
Day 1
Day 1
Day 1
Day 1
Day 5
Day 5
Day 9
Day 6
Day 9
Day 8
Day 5
post milrinone IA
pre milrinone
Day11
Day12
Day11
Day10
Day11
Day10
post milrinone IA
pre milrinone
post milrinone IA
pre milrinone
Thrombus post LIF

## Slide 9
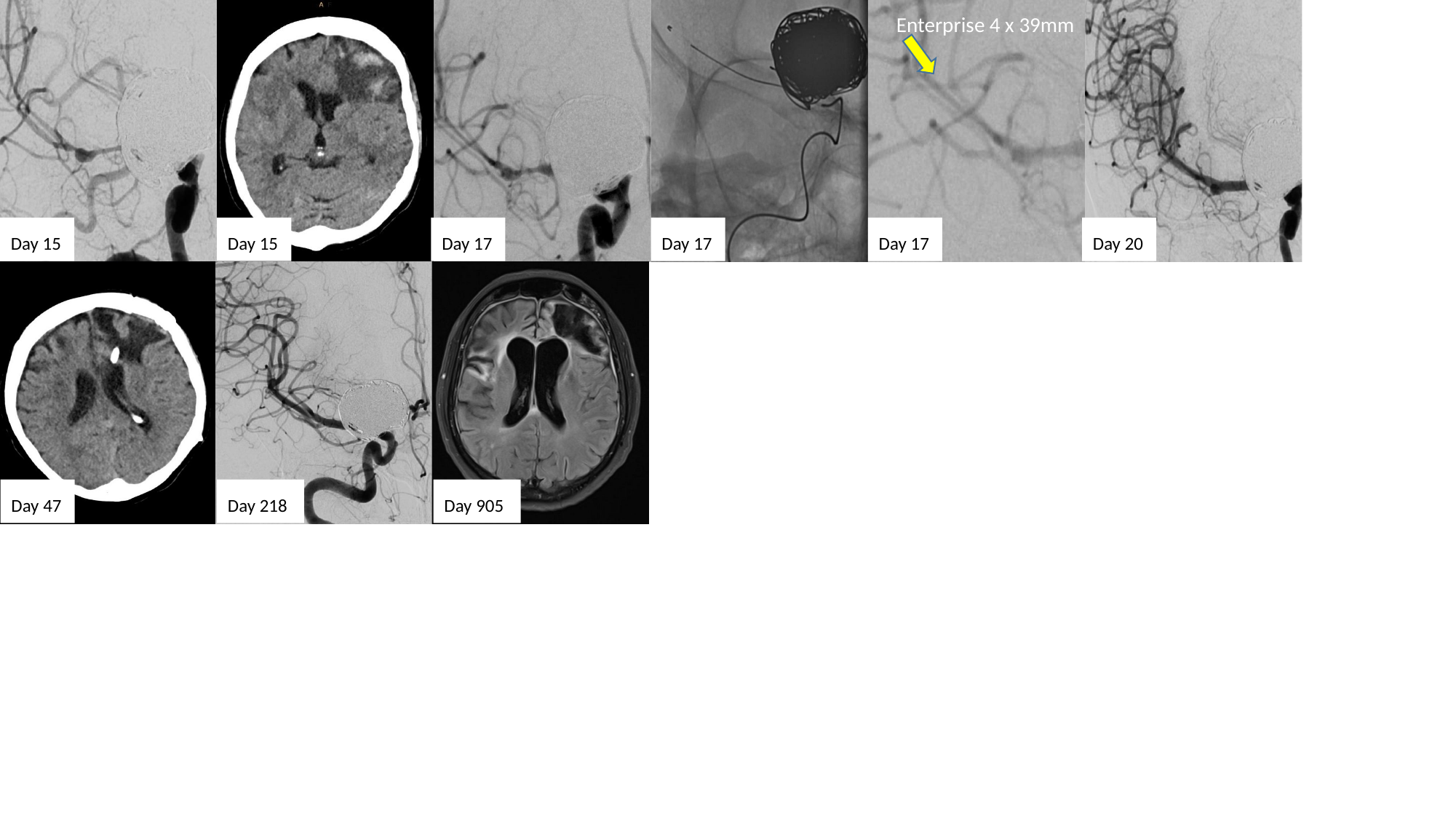

Enterprise 4 x 39mm
Day 15
Day 15
Day 17
Day 17
Day 17
Day 20
Day 47
Day 218
Day 905

## Slide 10
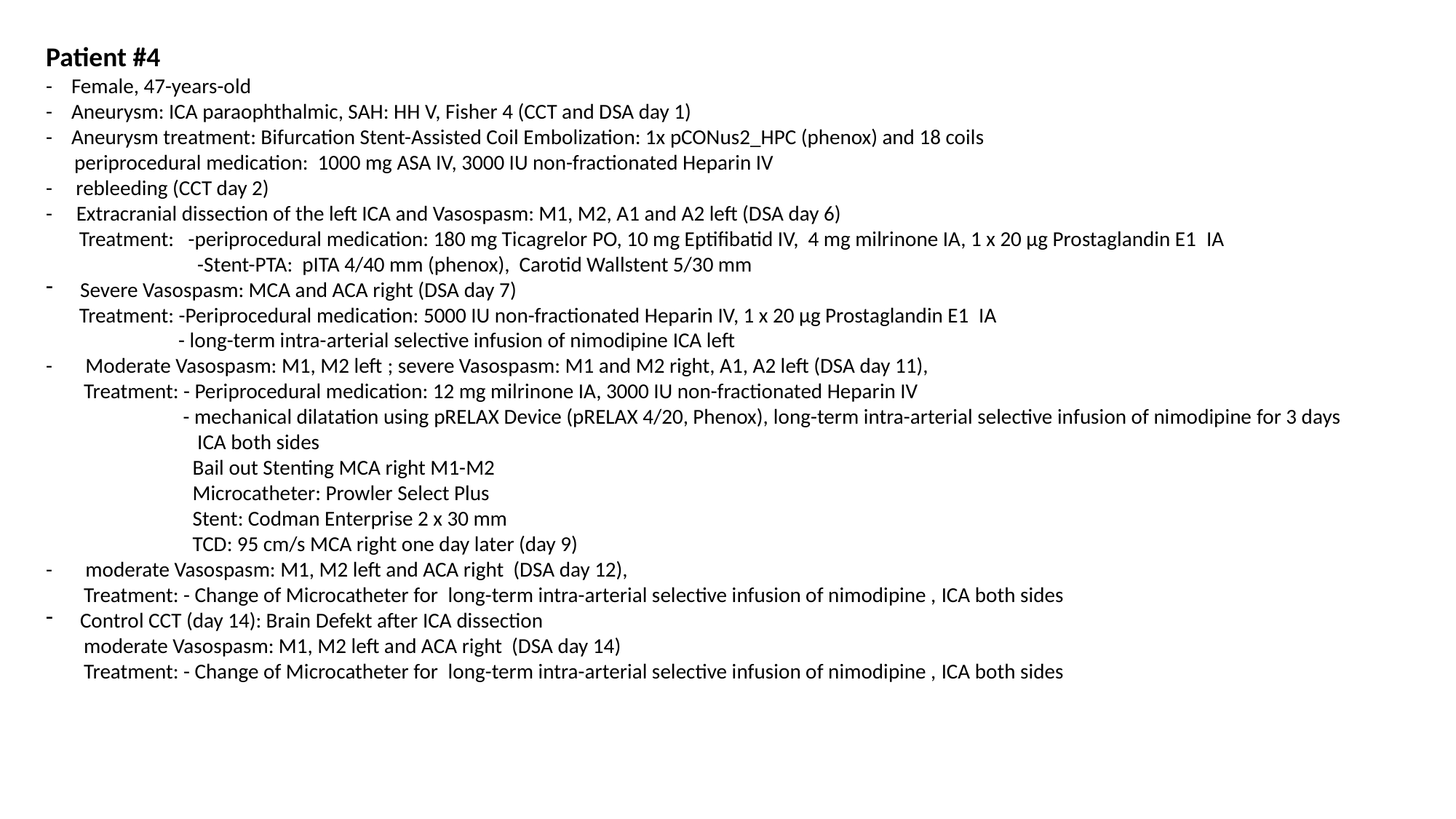

Patient #4
- Female, 47-years-old
- Aneurysm: ICA paraophthalmic, SAH: HH V, Fisher 4 (CCT and DSA day 1)
- Aneurysm treatment: Bifurcation Stent-Assisted Coil Embolization: 1x pCONus2_HPC (phenox) and 18 coils
 periprocedural medication: 1000 mg ASA IV, 3000 IU non-fractionated Heparin IV
- rebleeding (CCT day 2)
- Extracranial dissection of the left ICA and Vasospasm: M1, M2, A1 and A2 left (DSA day 6)
 Treatment: -periprocedural medication: 180 mg Ticagrelor PO, 10 mg Eptifibatid IV, 4 mg milrinone IA, 1 x 20 µg Prostaglandin E1  IA
 -Stent-PTA: pITA 4/40 mm (phenox), Carotid Wallstent 5/30 mm
Severe Vasospasm: MCA and ACA right (DSA day 7)
 Treatment: -Periprocedural medication: 5000 IU non-fractionated Heparin IV, 1 x 20 µg Prostaglandin E1  IA
 - long-term intra-arterial selective infusion of nimodipine ICA left
- Moderate Vasospasm: M1, M2 left ; severe Vasospasm: M1 and M2 right, A1, A2 left (DSA day 11),
 Treatment: - Periprocedural medication: 12 mg milrinone IA, 3000 IU non-fractionated Heparin IV
 - mechanical dilatation using pRELAX Device (pRELAX 4/20, Phenox), long-term intra-arterial selective infusion of nimodipine for 3 days
 ICA both sides
 Bail out Stenting MCA right M1-M2
 Microcatheter: Prowler Select Plus
 Stent: Codman Enterprise 2 x 30 mm
 TCD: 95 cm/s MCA right one day later (day 9)
- moderate Vasospasm: M1, M2 left and ACA right (DSA day 12),
 Treatment: - Change of Microcatheter for long-term intra-arterial selective infusion of nimodipine , ICA both sides
Control CCT (day 14): Brain Defekt after ICA dissection
 moderate Vasospasm: M1, M2 left and ACA right (DSA day 14)
 Treatment: - Change of Microcatheter for long-term intra-arterial selective infusion of nimodipine , ICA both sides

## Slide 11
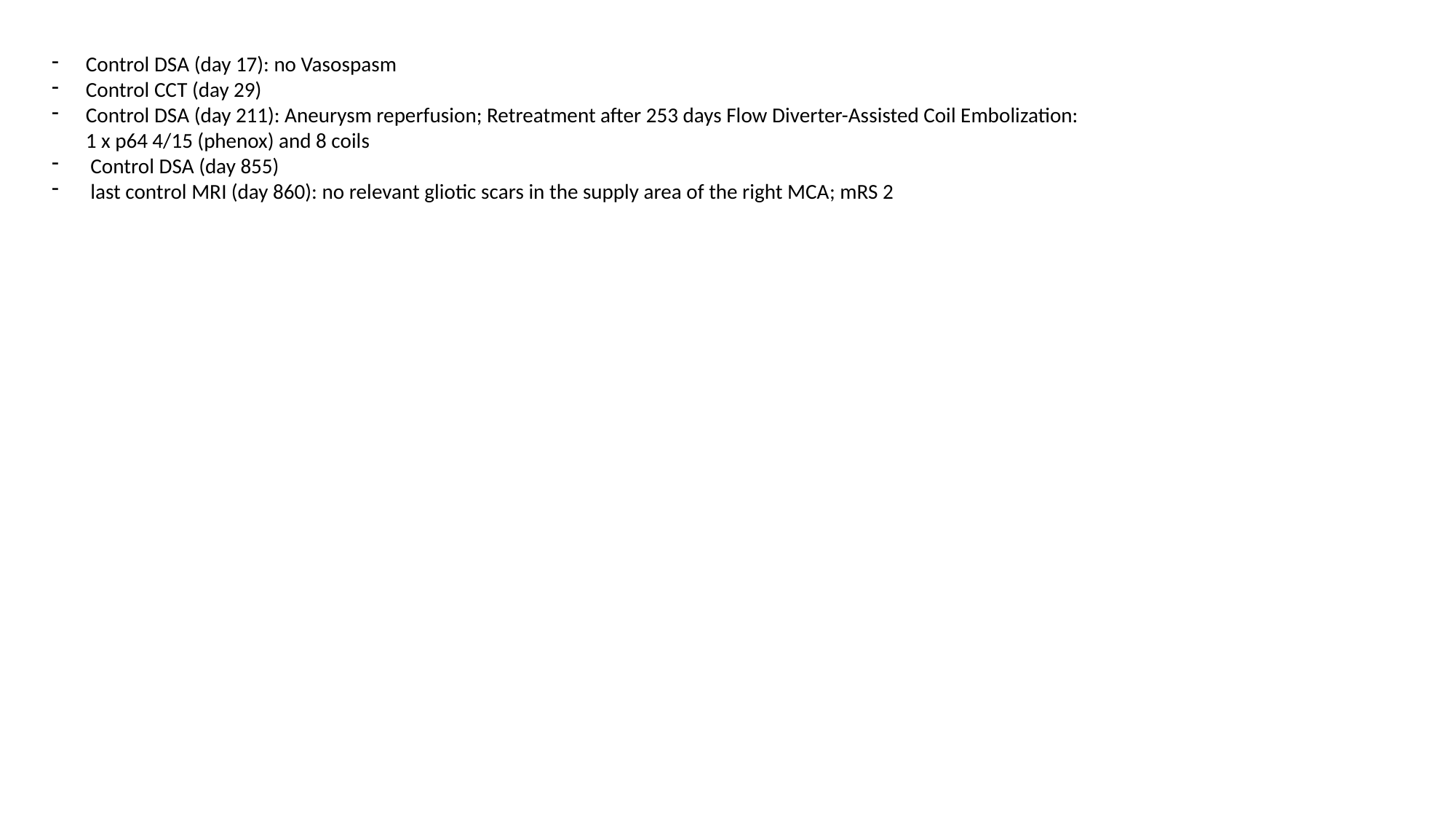

Control DSA (day 17): no Vasospasm
Control CCT (day 29)
Control DSA (day 211): Aneurysm reperfusion; Retreatment after 253 days Flow Diverter-Assisted Coil Embolization: 1 x p64 4/15 (phenox) and 8 coils
 Control DSA (day 855)
 last control MRI (day 860): no relevant gliotic scars in the supply area of the right MCA; mRS 2

## Slide 12
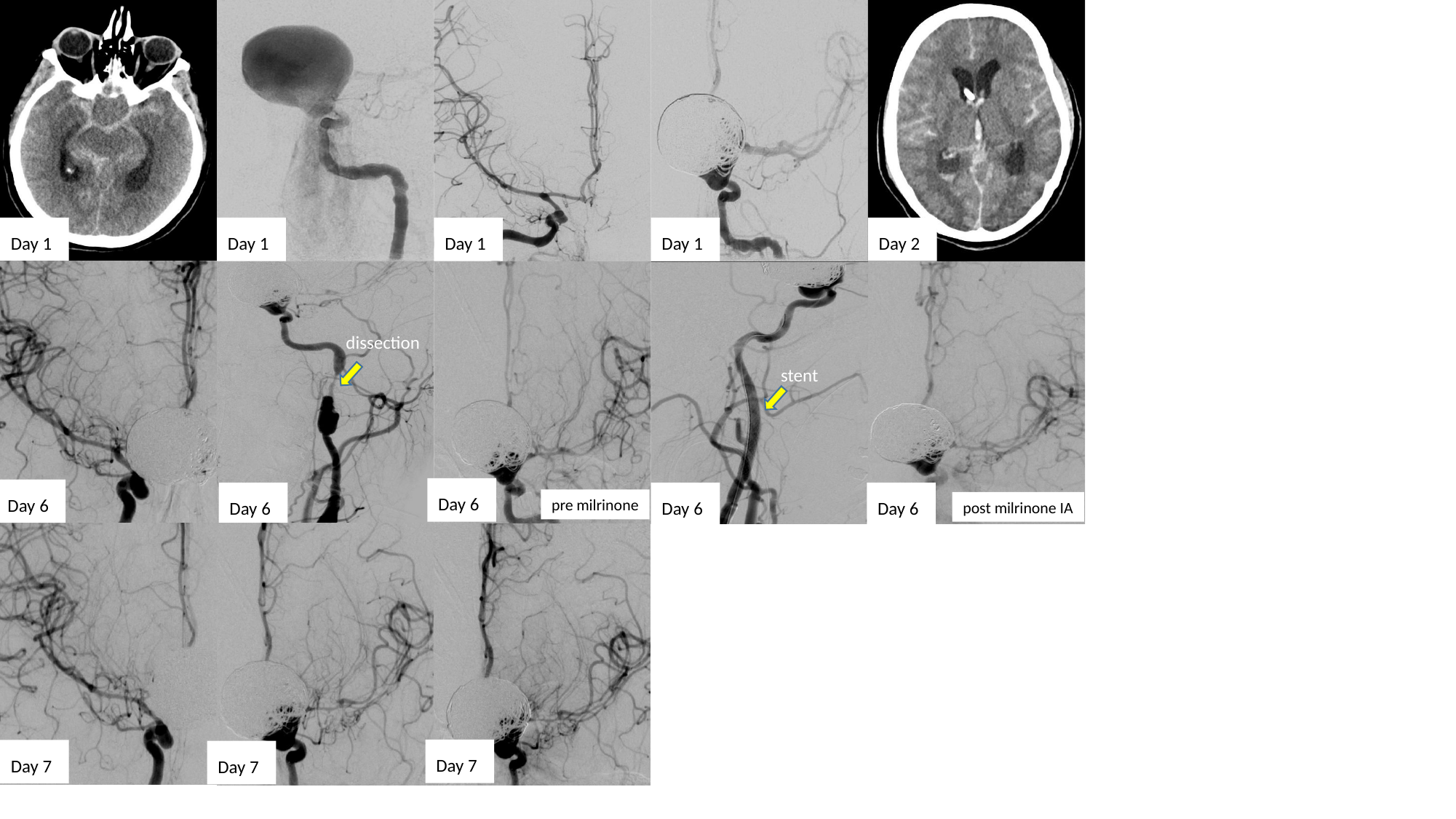

Day 1
Day 1
Day 1
Day 1
Day 2
dissection
stent
Day 6
Day 6
Day 6
Day 6
Day 6
pre milrinone
post milrinone IA
Day 7
Day 7
Day 7
Thrombus post LIF

## Slide 13
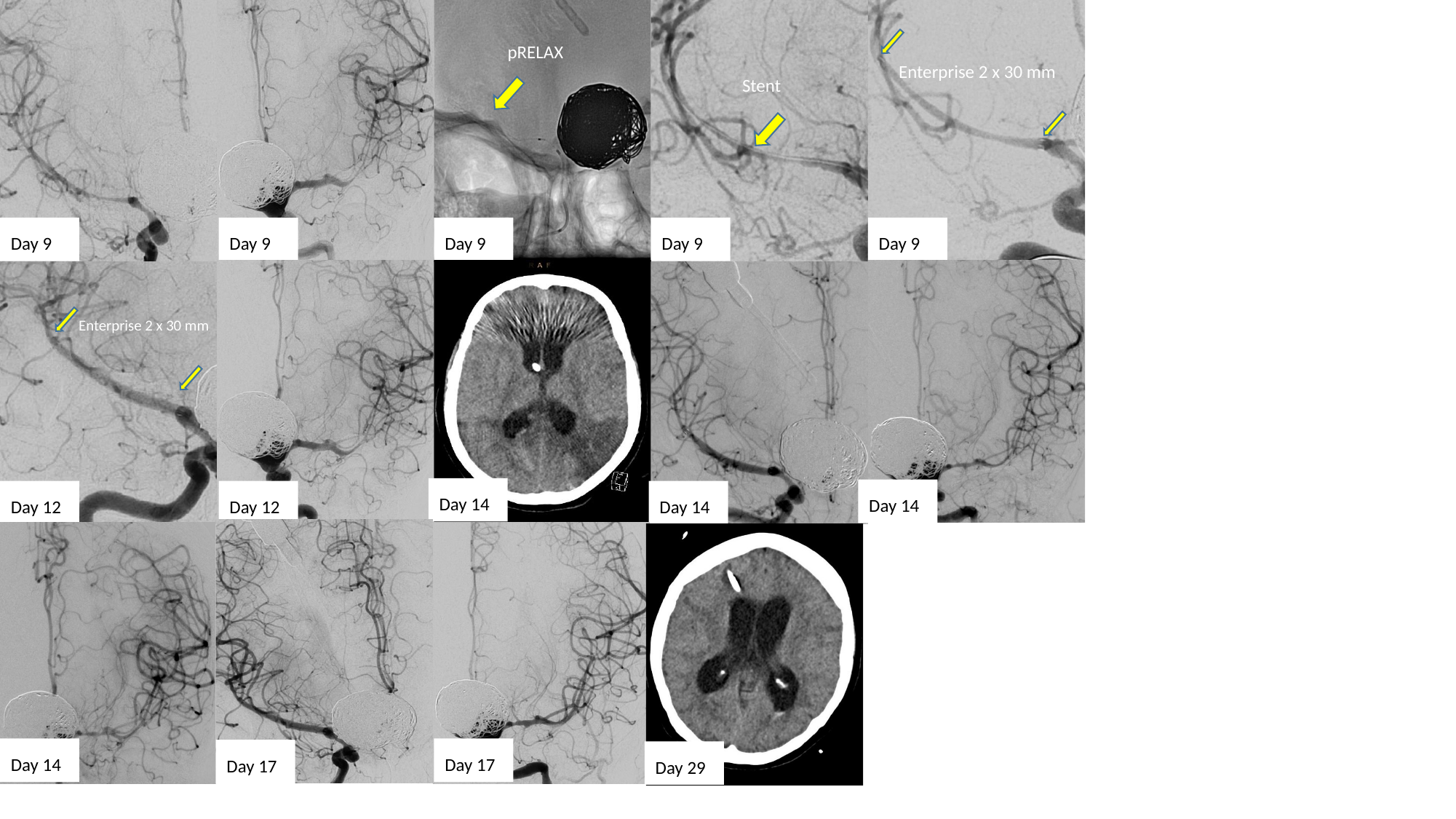

pRELAX
 Enterprise 2 x 30 mm
Stent
Day 9
Day 9
Day 9
Day 9
Day 9
 Enterprise 2 x 30 mm
Day 14
Day 14
Day 12
Day 12
Day 14
Day 14
Day 17
Day 17
Day 29

## Slide 14
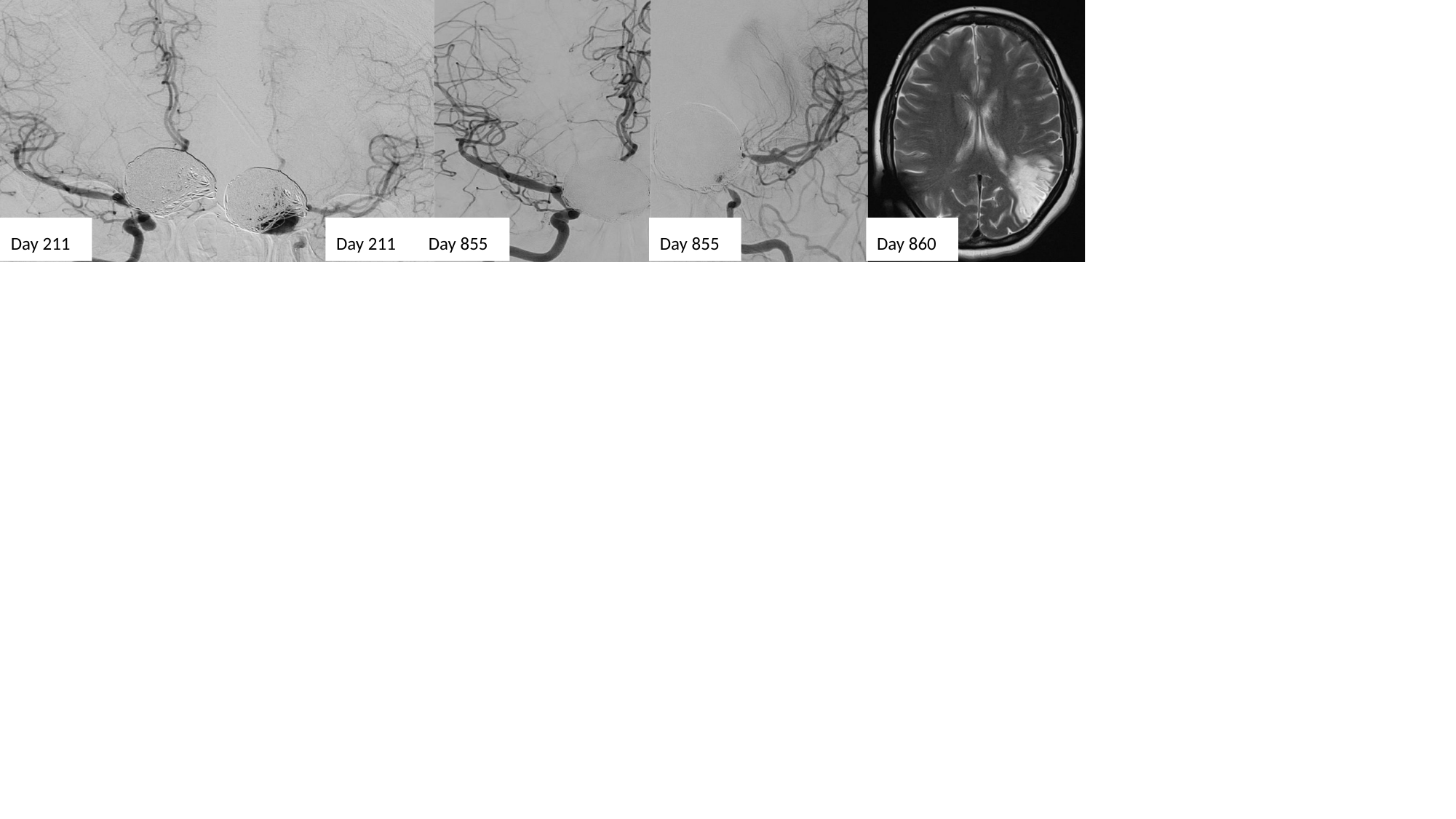

Day 211
Day 211
Day 855
Day 855
Day 860

## Slide 15
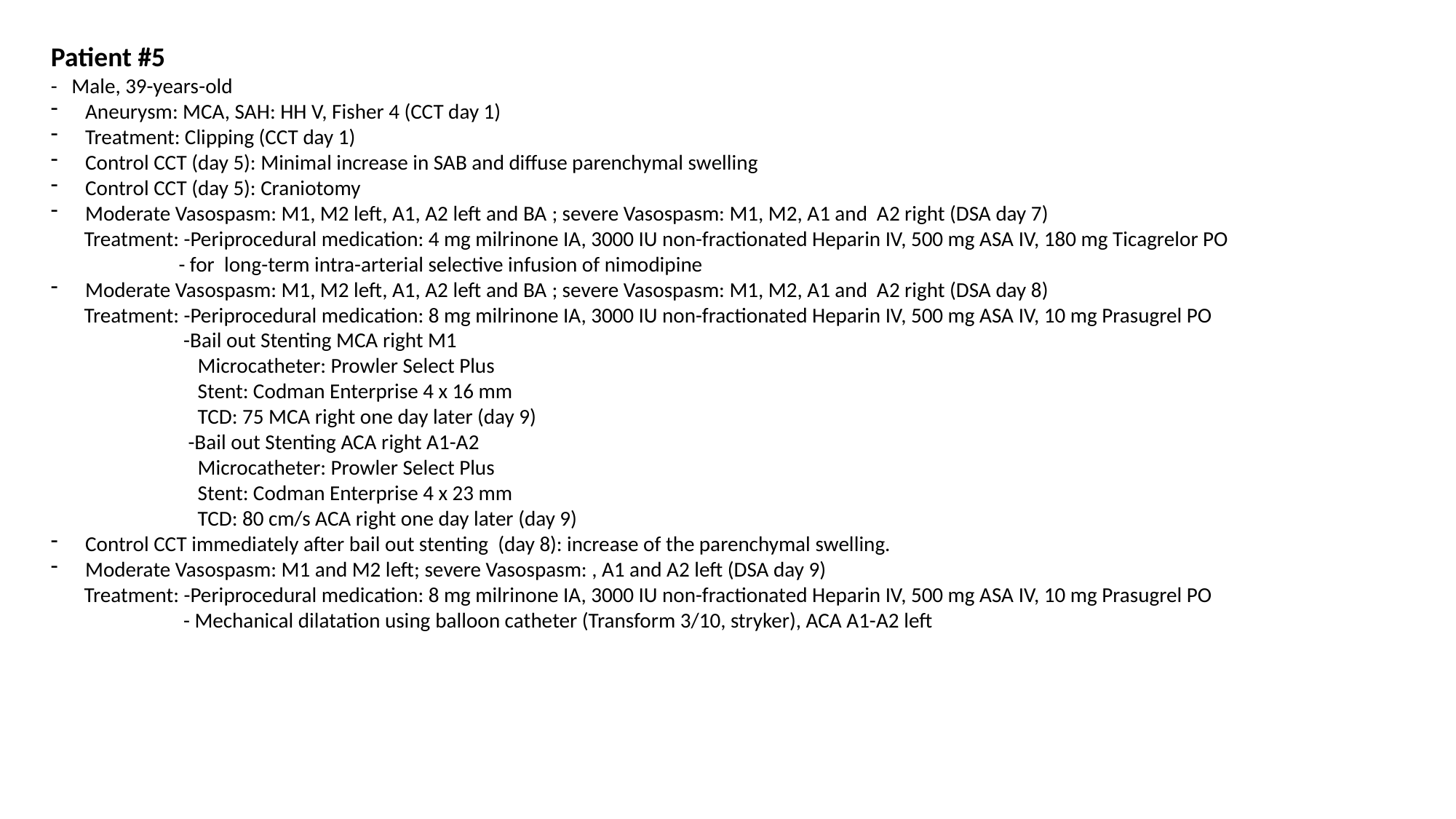

Patient #5
- Male, 39-years-old
Aneurysm: MCA, SAH: HH V, Fisher 4 (CCT day 1)
Treatment: Clipping (CCT day 1)
Control CCT (day 5): Minimal increase in SAB and diffuse parenchymal swelling
Control CCT (day 5): Craniotomy
Moderate Vasospasm: M1, M2 left, A1, A2 left and BA ; severe Vasospasm: M1, M2, A1 and A2 right (DSA day 7)
 Treatment: -Periprocedural medication: 4 mg milrinone IA, 3000 IU non-fractionated Heparin IV, 500 mg ASA IV, 180 mg Ticagrelor PO
 - for long-term intra-arterial selective infusion of nimodipine
Moderate Vasospasm: M1, M2 left, A1, A2 left and BA ; severe Vasospasm: M1, M2, A1 and A2 right (DSA day 8)
 Treatment: -Periprocedural medication: 8 mg milrinone IA, 3000 IU non-fractionated Heparin IV, 500 mg ASA IV, 10 mg Prasugrel PO
 -Bail out Stenting MCA right M1
 Microcatheter: Prowler Select Plus
 Stent: Codman Enterprise 4 x 16 mm
 TCD: 75 MCA right one day later (day 9)
 -Bail out Stenting ACA right A1-A2
 Microcatheter: Prowler Select Plus
 Stent: Codman Enterprise 4 x 23 mm
 TCD: 80 cm/s ACA right one day later (day 9)
Control CCT immediately after bail out stenting (day 8): increase of the parenchymal swelling.
Moderate Vasospasm: M1 and M2 left; severe Vasospasm: , A1 and A2 left (DSA day 9)
 Treatment: -Periprocedural medication: 8 mg milrinone IA, 3000 IU non-fractionated Heparin IV, 500 mg ASA IV, 10 mg Prasugrel PO
 - Mechanical dilatation using balloon catheter (Transform 3/10, stryker), ACA A1-A2 left

## Slide 16
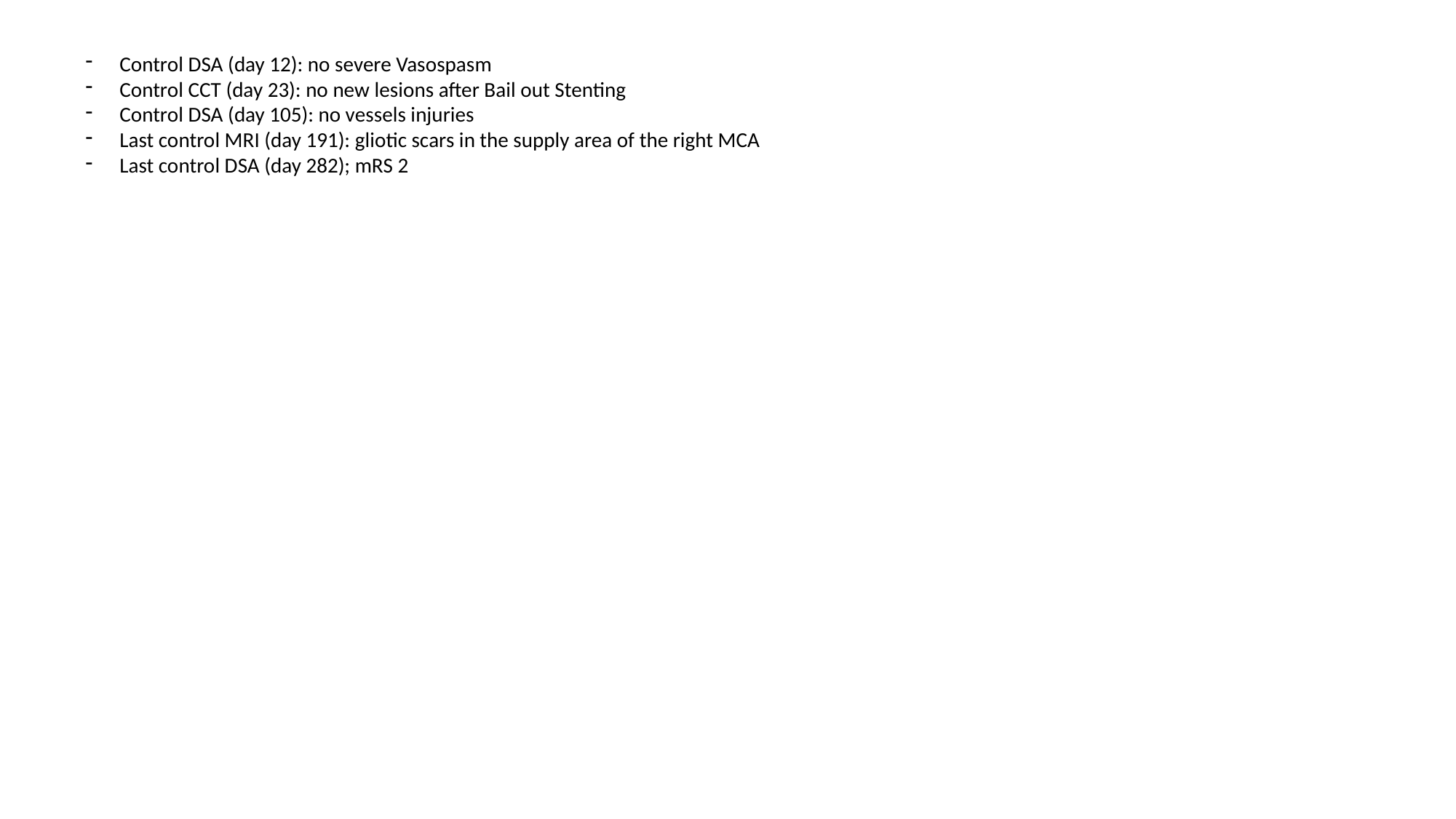

Control DSA (day 12): no severe Vasospasm
Control CCT (day 23): no new lesions after Bail out Stenting
Control DSA (day 105): no vessels injuries
Last control MRI (day 191): gliotic scars in the supply area of the right MCA
Last control DSA (day 282); mRS 2

## Slide 17
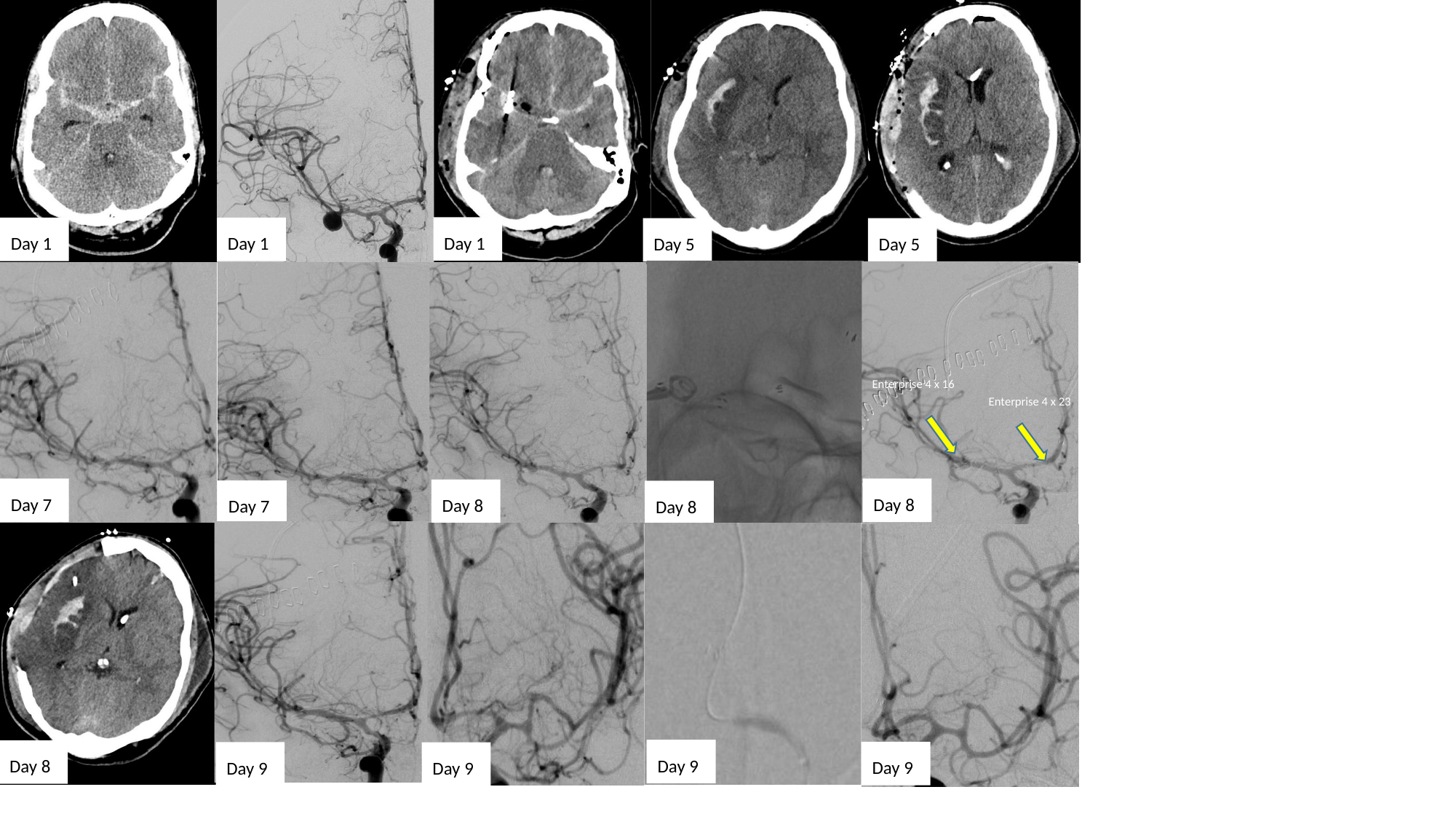

Day 1
Day 1
Day 1
Day 5
Day 5
 Enterprise 4 x 16
 Enterprise 4 x 23
Day 7
Day 8
Day 8
Day 7
Day 8
Day 9
Day 8
Day 9
Day 9
Day 9
Day 9
Thrombus post LIF

## Slide 18
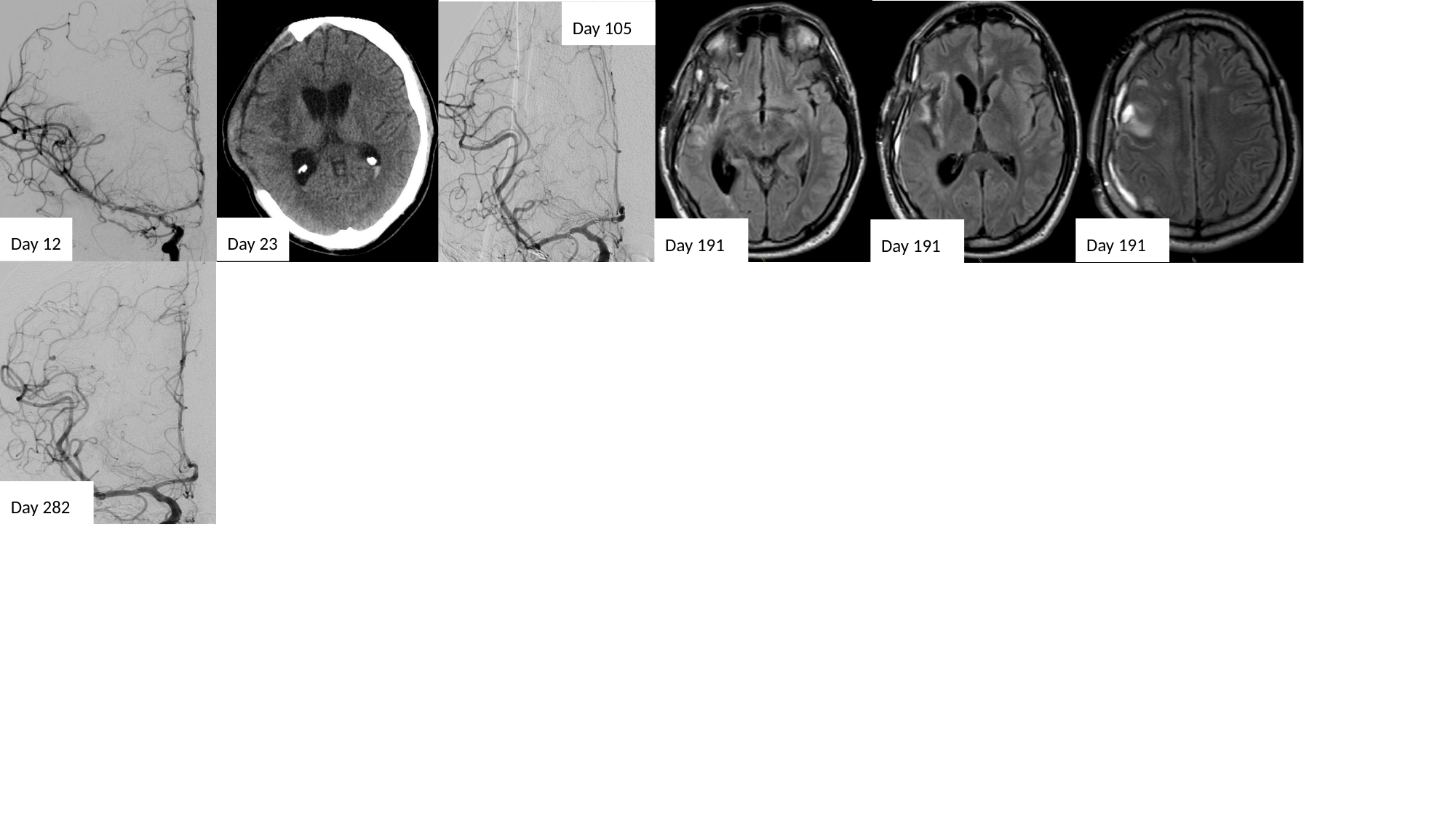

Day 105
Day 12
Day 23
Day 191
Day 191
Day 191
Day 282

## Slide 19
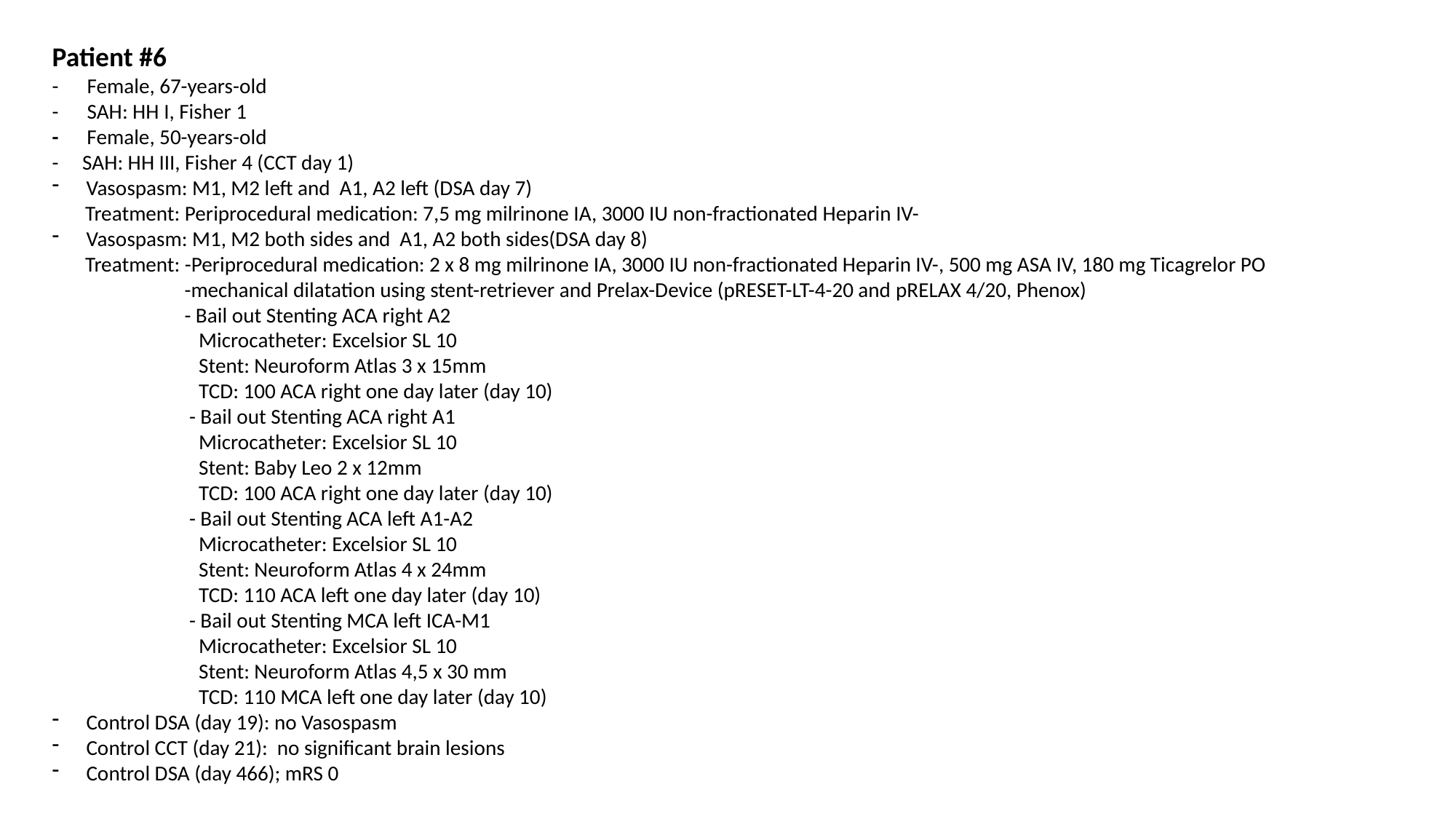

Patient #6
- Female, 67-years-old
- SAH: HH I, Fisher 1
- Female, 50-years-old
- SAH: HH III, Fisher 4 (CCT day 1)
Vasospasm: M1, M2 left and A1, A2 left (DSA day 7)
 Treatment: Periprocedural medication: 7,5 mg milrinone IA, 3000 IU non-fractionated Heparin IV-
Vasospasm: M1, M2 both sides and A1, A2 both sides(DSA day 8)
 Treatment: -Periprocedural medication: 2 x 8 mg milrinone IA, 3000 IU non-fractionated Heparin IV-, 500 mg ASA IV, 180 mg Ticagrelor PO
 -mechanical dilatation using stent-retriever and Prelax-Device (pRESET-LT-4-20 and pRELAX 4/20, Phenox)
 - Bail out Stenting ACA right A2
 Microcatheter: Excelsior SL 10
 Stent: Neuroform Atlas 3 x 15mm
 TCD: 100 ACA right one day later (day 10)
 - Bail out Stenting ACA right A1
 Microcatheter: Excelsior SL 10
 Stent: Baby Leo 2 x 12mm
 TCD: 100 ACA right one day later (day 10)
 - Bail out Stenting ACA left A1-A2
 Microcatheter: Excelsior SL 10
 Stent: Neuroform Atlas 4 x 24mm
 TCD: 110 ACA left one day later (day 10)
 - Bail out Stenting MCA left ICA-M1
 Microcatheter: Excelsior SL 10
 Stent: Neuroform Atlas 4,5 x 30 mm
 TCD: 110 MCA left one day later (day 10)
Control DSA (day 19): no Vasospasm
Control CCT (day 21): no significant brain lesions
Control DSA (day 466); mRS 0
Periprocedural Vasospasm: M1, M2 right, coil protrusion
Microcatheter: Headway17
Stent: LVIS Jr; M2 rt inferior trunk (Ø 0.9 mm) => M1 right (Ø 1.2 mm)
Periprocedural medication: 8 mg milrinone IA, 2x 500 mg ASA IV, 600 mg Clopidogrel PO, 3000 IU non-fractionated Heparin IV,
4 mg eptifibatide IA
Postprocedural medication: 100 mg ASA PO daily for life, 75 mg Clopidogrel PO daily for 6 months
Nimotop IV: from day 5 through day ##
TCD: day #: vessel, value, day #: vessel, value, day #: vessel, value, day #: vessel, value
No additional vasospasm treatment required
MRI on day ### after the SAH showed gliotic scars of the right frontal lobe;
Clinical outcome: mRS 0, #### days after the SAH (telephone interview)

## Slide 20
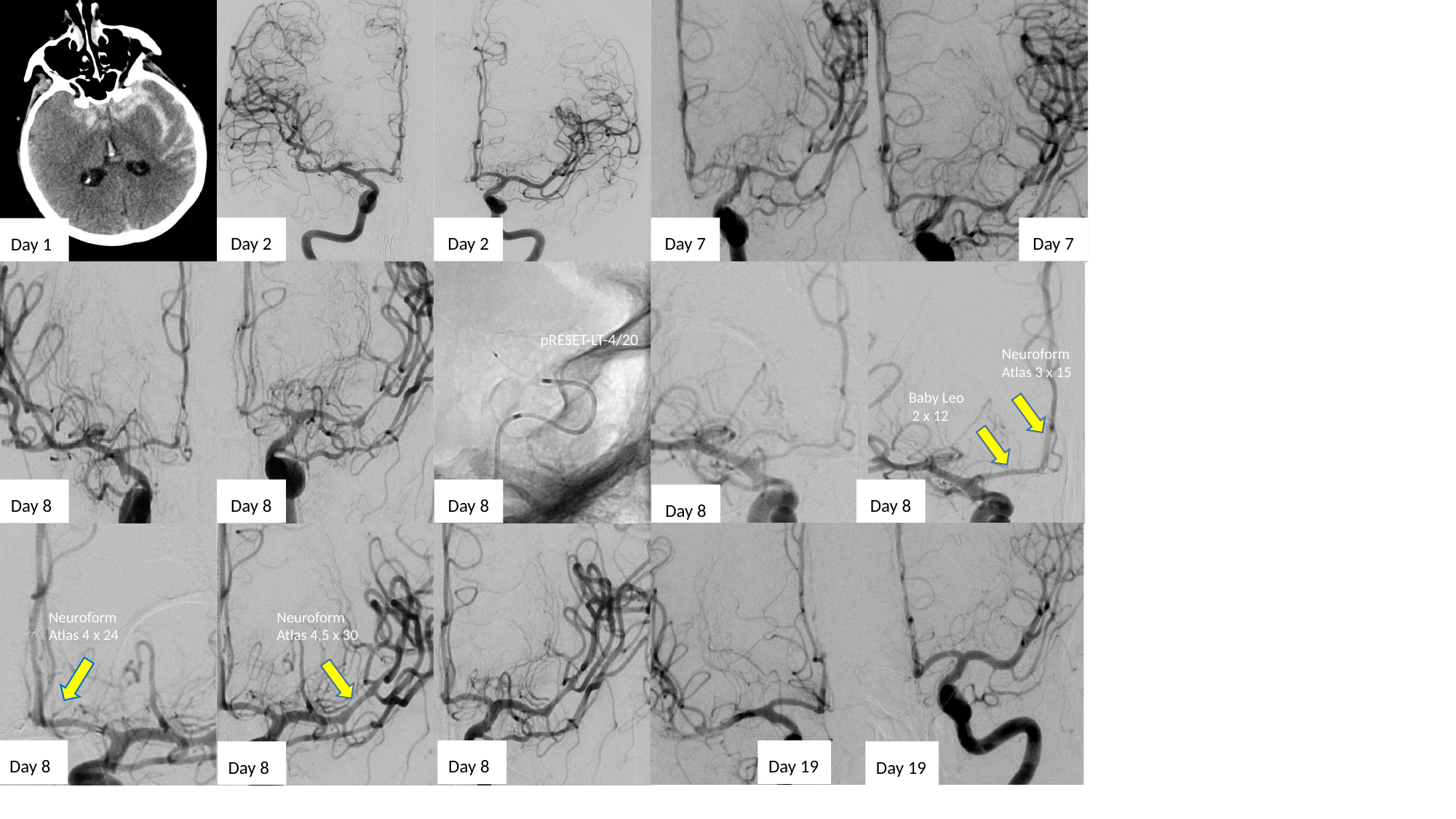

Day 2
Day 2
Day 7
Day 7
Day 1
pRESET-LT-4/20
Neuroform Atlas 3 x 15
Baby Leo
 2 x 12
Day 8
Day 8
Day 8
Day 8
Day 8
Neuroform Atlas 4 x 24
Neuroform Atlas 4,5 x 30
Day 8
Day 8
Day 19
Day 8
Day 19
Thrombus post LIF

## Slide 21
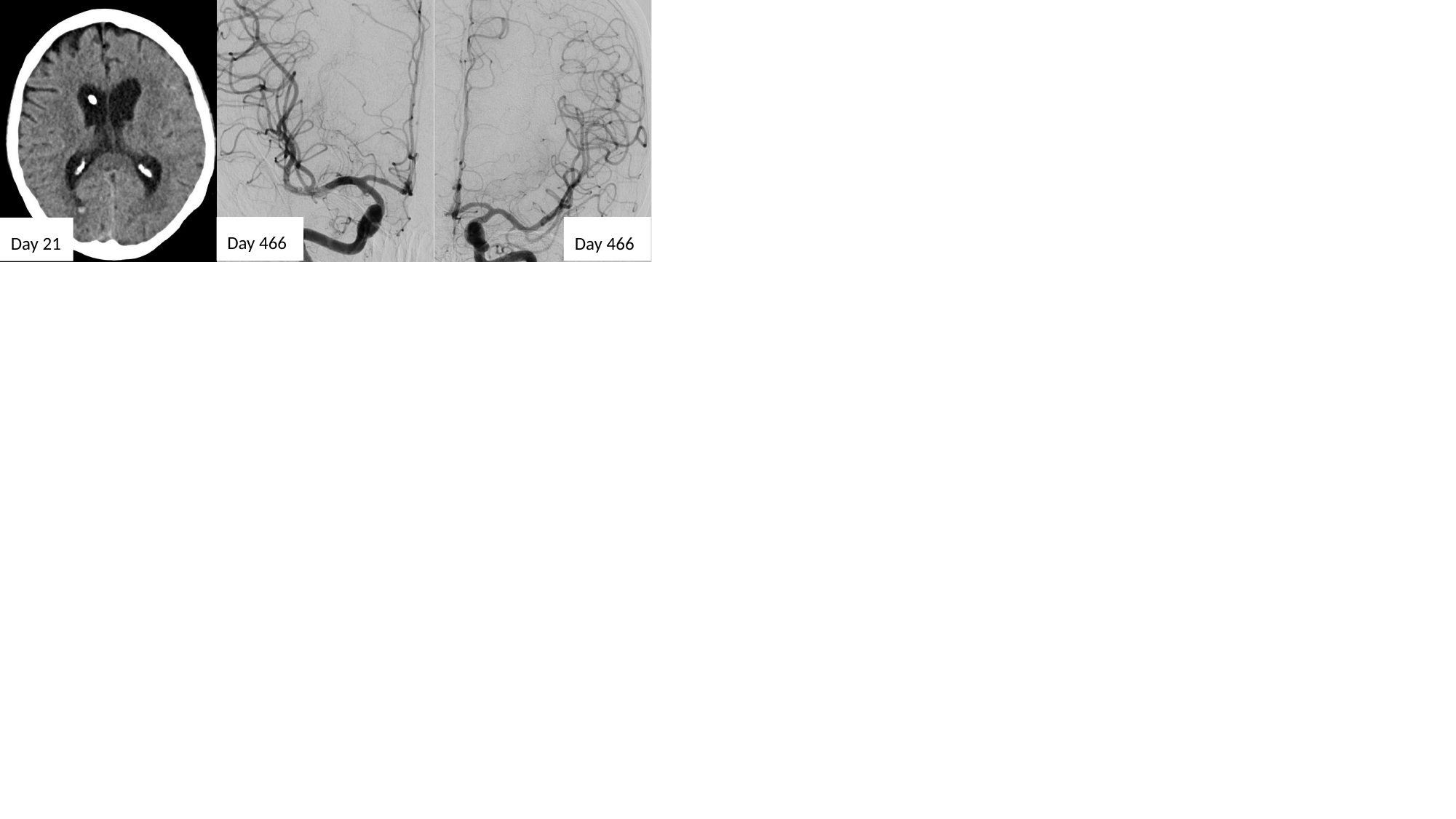

Day 466
Day 466
Day 21
